# Fugitive Dust Associated with Scrap Metal Processing

**DOI:** 10.3390/environments10120223

**Published:** 2023-12-14

**Authors:** Jeff Gearhart, Simone Sagovac, Tian Xia, Md Kamrul Islam, Albert Shim, Sung-Hee Seo, Melissa Cooper Sargent, Natalie R. Sampson, Jacob Napieralski, Ika Danielson, Stuart Batterman

**Affiliations:** 1 Ecology Center, Ann Arbor, MI 48104, USA; 2 Southwest Detroit Community Benefits Coalition, Detroit, MI 48209, USA; 3 Department of Environmental Health Sciences, University of Michigan, Ann Arbor, MI 48109, USA; 4 Department of Natural Sciences, and Health and Human Services, University of Michigan, Dearborn, MI 48128, USA

**Keywords:** fugitive dust, metal processors, iron, copper, chromium, detroit, environmental justice, particulate matter

## Abstract

Fugitive dust (FD) is a nuisance and potential health issue, particularly in environmental justice communities that can experience high levels of contaminated FD. This community-initiated study examined FD from a scrap metal processor in Detroit, Michigan, to determine whether the FD was contaminated, how it migrated through the community, whether wipe or composite road dust samples were preferable, and whether literature profiles adequately characterized this source. The study was motivated by community concerns, as well as a massive subsidence/upheaval event resulting from excessive accumulation of mill scale, which is a type of scrap metal, at the facility. We collected 57 wipe samples from windows and other surfaces, and 20 composite road dust and surface soil samples, which were analyzed by X-ray fluorescence. Concentrations were expressed using the fraction of the reconstructed mass. We also compared results to air quality monitoring data and calculated pollution indices and enrichment factors. Samples collected near the processor had high levels of Cr, Cu, Fe, Ni, Sr, and Zn compared with background soils, and levels remained elevated in residential areas several blocks distant. Composite road dust/sediment samples appeared preferable to wipe samples for chemical characterization. The available chemical profiles did not match the FD composition, suggesting the need for local profiles. The high level of Fe, which is consistent with mill scale, was a novel finding and caused the road dust to exceed health protection screening levels. Numerous metal scrap facilities operate locally and nationally, and our results show the need to improve controls to limit or eliminate FD emissions from industrial sources using enforced policies that reduce dust generation and truck track-out.

## 1. Introduction

Fugitive dust (FD) is particulate matter that enters the air from non-stack sources [1]. Common sources of FD include soil and road dust (e.g., vehicle- and wind-entrained silt, road, tire and brake wear, street repair and cleaning), construction dust (concrete cutting, truck track-out), industry (material storage, processing, transfer) and agriculture (entrained soil, grain handling). Much of FD is “coarse” size fraction particles (aerodynamic diameter between 2.5–10 μm) and “large” coarse particles (>10 μm) that are deposited near the source due to sedimentation and called dustfall. FD emissions can be controlled using basic housekeeping measures and appropriate street cleaning practices, and for industrial sources, the use of enclosed or covered storage, transfer and transport systems; fences and windscreens; limits on open piles, e.g., maximum heights or buffers; and operational restrictions, e.g., maximum drop heights and wind speed limits for material handling operations [1]. Despite such controls, FD is often a long-standing nuisance and potential health issue that receives little attention, which can be attributed to several factors: its relatively modest contribution to PM_2.5_, which is typically the pollutant of most health concern; the limited monitoring of larger-sized particulate matter (e.g., PM_10_ and total suspended particulate (TSP)); the abandonment of urban deposition and dustfall measurements; the localized impacts from individual sources (since large particles rapidly settle to the ground); the perception that soiling and dust accumulation are only aesthetic concerns; and the perception that emissions are transitory, uncommon, and must be tolerated due to short-term activities, such as construction. Additional reasons, including the designation of high FD levels as “exceptional events” that are excluded from regulatory consideration, which is especially relevant in rural areas, have been suggested [2]. While the U.S. Environmental Protection Agency (EPA) estimates FD emissions as part of PM_10_ at the county level [3], assessments of localized impacts require finer spatial resolution and should include ultra-coarse PM (diameter >10 μm), which is an important limitation given its large contribution to FD, e.g., 60% of the total FD from unpaved roads [4].

Soil in urban areas tends to accumulate trace elements compared with outlying areas [5], and studies across the world showed elevated levels of Pb, Zn, Cu, Cr, Ni, and other contaminants in urban soils [6–8]. For example, a review of urban soil studies conducted in 1979–2018 had elevated pollution indices (PIs), which show the impact of local contamination sources. The PI is defined as the ratio of concentrations in the urban area relative to “background” levels estimated using samples collected in non-urban or comparison areas (distant from sources of concern). The PI exceeded 5 for Cd, Cu, Ni, and Pb, which was deemed an extremely high level of pollution, and was lower but still elevated for Cr and Zn, which was called moderately to highly polluted [9]. In communities experiencing environmental injustices, residential neighborhoods often are adjacent to ongoing or past industrial activity that can contaminate soils [10]. For example, in Santa Ana, California, 90% of 1500 soil samples exceeded EPA screening levels for As and Ni (using a hazard index of 0.1), and Zn and Pb levels were especially high in low-income areas [11]. In Detroit, Michigan, high levels of Cr (198 ppm) and Pb (134 ppm) were found in soil and road dust [12]. In Pueblo, Colorado, a community with a history of industry and agriculture, the As, Cd, Hg, and Pb levels were the highest in low-income, minority communities [13]. In a review of 10 European studies, lower socioeconomic status residents experienced greater exposure to FD associated with proximity to mining, incinerators, and landfills [14]. Such studies demonstrate that communities burdened by cumulative environmental exposures also may experience high levels of contaminated FD, leading to the potential of exposure and further risk of adverse health impacts.

The present study investigates the composition and dispersal of FD in a community situated around a specific industrial source, namely, a scrap metal processor. Our goals were to determine whether the FD associated with the facility was contaminated, assess whether the FD had a unique composition or “profile” that could be tracked through the community, compare wipe samples with composite road dust and soil samples, evaluate whether new profiles are needed to identify and characterize specific FD sources, assess the need for “hyperlocal” investigations for FD sources, and begin to characterize the risk to inform advocacy efforts toward environmental justice. The study was motivated by community concerns regarding FD generated and entrained by haulage trucks to the facility, despite suppression efforts entailing street cleaning and wetting and some housekeeping activities at the processor.

## 2. Materials and Methods

### 2.1. Case Study Facility

**Figure 1.**
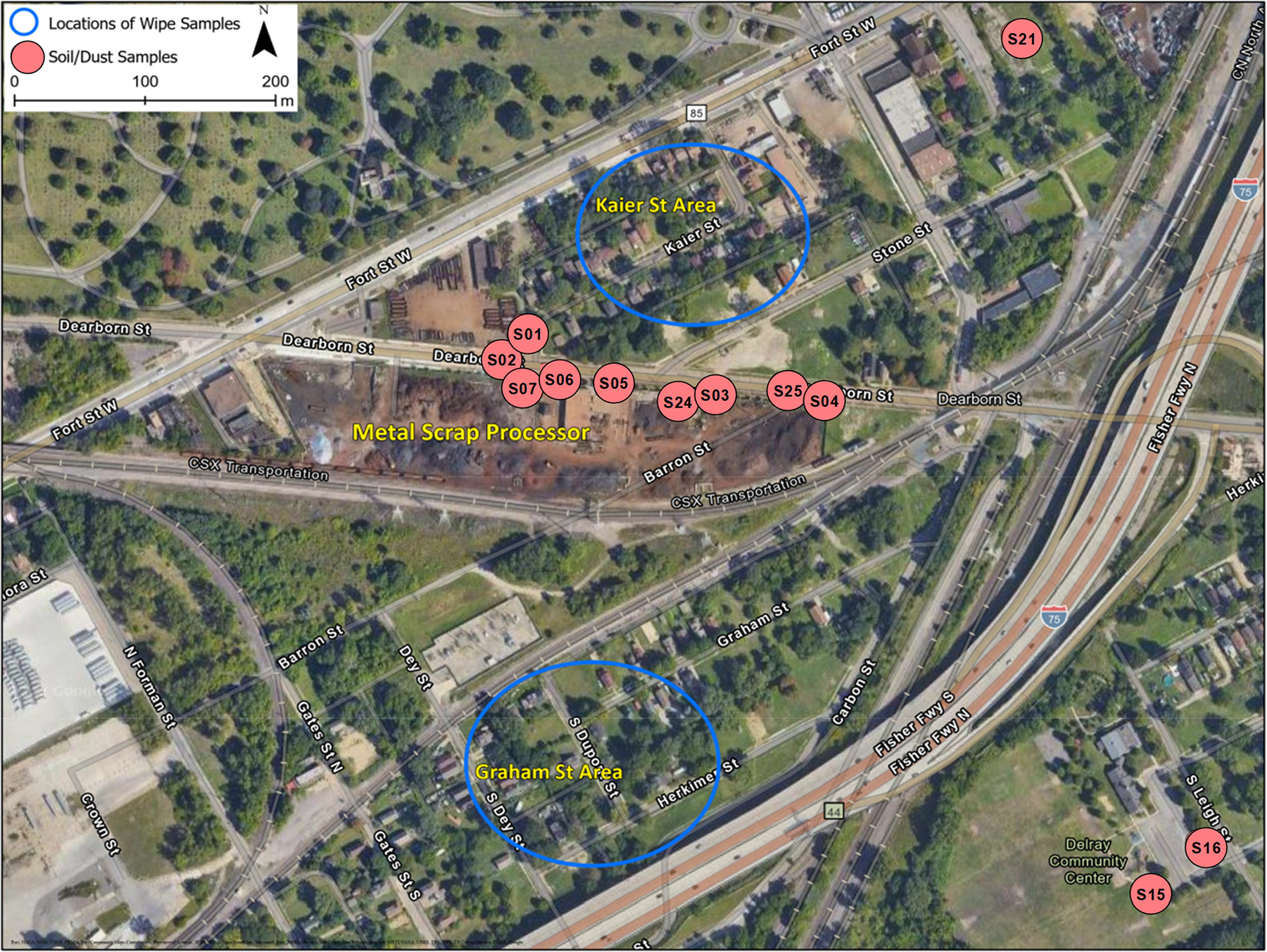
Aerial view around the metal processor in southwest Detroit showing locations of road dust/soil samples. Wipe samples were collected in blue-circled areas in the Kaier and Graham Street zones.

**Figure 2.**
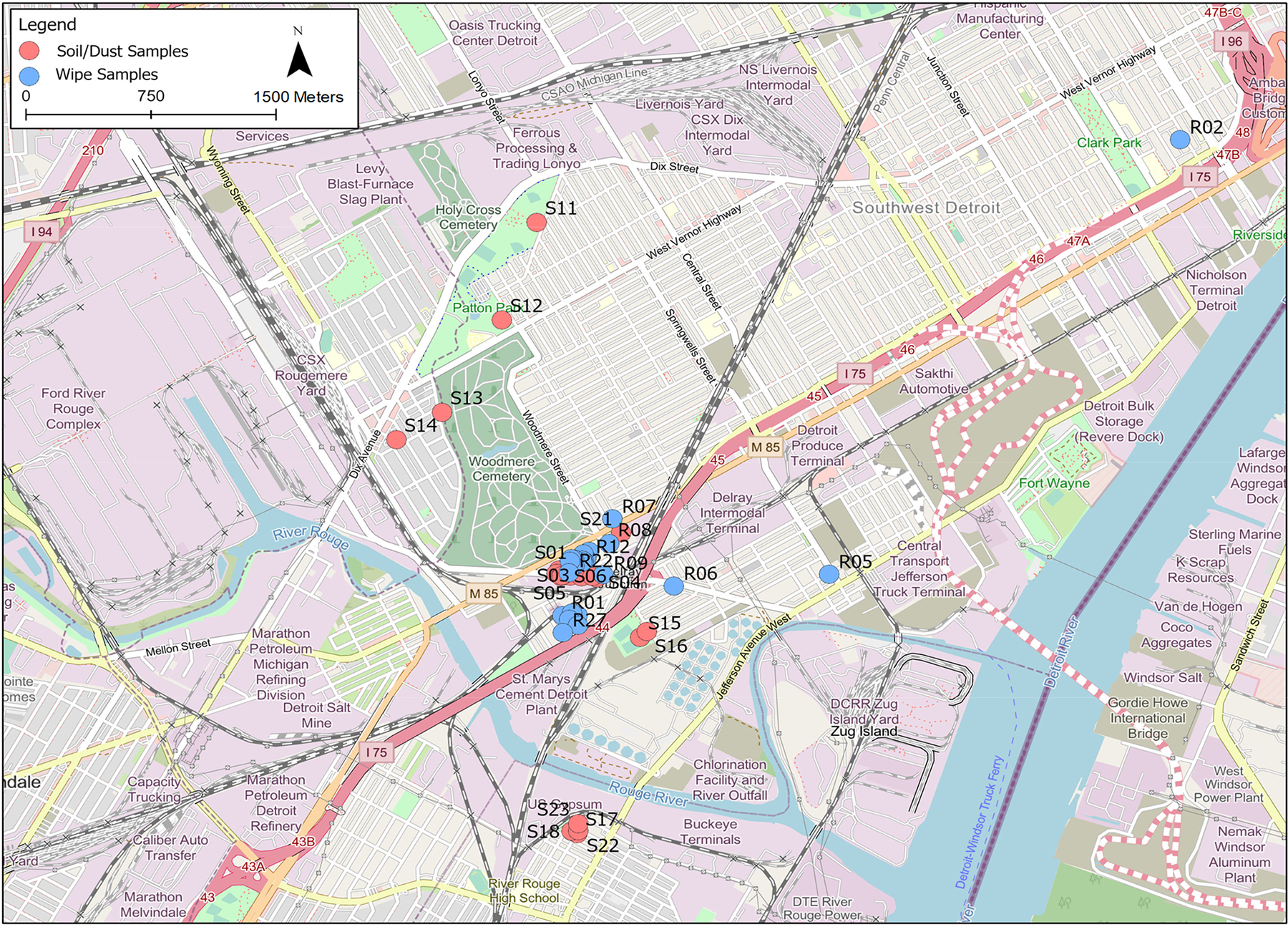
Street map showing larger study region and outlying sampling sites.

The facility of concern collects and stores metal scrap and provides basic separation and sizing (e.g., mechanical and torch cutting), bulking, and shipment off site. It is located in Southwest Detroit, Michigan, a community of approximately 60,000 persons that has been industrialized for well over a century. The processor has operated since the mid-to-late 1980s and has been in its present configuration since about 2002 based on aerial photos (Figure S1 in the supplemental information (SI) shows a 1949 aerial view; figures and tables in the SI are noted with a prefix “S”). Figures 1 and 2 show aerial views of the facility and the larger area, respectively. Photographs of the operations at the site are shown in Figure S2.

The 3.5 ha facility is unpaved, surrounded by a ~2 m high solid fence on three sides and has a frontage of 438 m along Dearborn Street on the north side. Heavy equipment (10–20 loaders, conveyers, dump trucks) operates on site, based on visual inspection and aerial images. Haulage trucks enter from Dearborn Street via Fort Street, and material leaves the facility via a rail siding at the property’s south side. During working hours, we estimated that roughly 12 haul trucks entered or exited the facility each hour, representing a subset of trucks (34–53 trucks/hr) observed on Dearborn Street. The estimated total and commercial (truck) vehicle volumes on Fort Street were 7551 and 945 vehicles per day [15]. Residential communities lay immediately to the north on Kaier and adjoining streets, and to the south on Graham and other streets (Figure 1). Areas to the north on Dearborn Street also include an auto repair shop, a dumpster storage yard, and other residential areas.

The metal processor attained notoriety on 11 September 2021. After what appeared to be a several-year accumulation of mill scale (fine-grained mixed iron oxides that include FeO, Fe_2_O_3_, and Fe_3_O_4_) in a pile that reached 25 feet above grade collected from two nearby steel mills, the pile’s weight coupled with unusually high rainfall and soft underlying clay soils led to bearing capacity failure. The subsidence beneath the pile caused the surrounding soils to heave upward by up to 7 ft [16], affecting adjacent properties and the intersection of Dearborn and Fort Streets, where a 16-inch diameter water main ruptured, local property was damaged and a fire ignited that burned down an adjacent building. This event caused a ~1-month shutdown of Fort Street to repair utilities and reconstruct the road and raised safety concerns among residents due to initial confusion about the cause of the upheaval, appropriate emergency responses, and issues with disrupted gas and water services. Over the following year, the mill scale was removed from the facility.

### 2.2. Environmental Sampling

In September 2022 and prior to sampling, the community–academic team hosted an event in the neighborhood near the scrapyard to engage residents about the issue of FD, let them know about future sampling, and discuss any questions or concerns about the dust sampling.

Road dust/sediment, soil, and wipe samples were collected in several zones: near the metal processor on Dearborn Street, at nearby residential areas north and south of the processor (i.e., Kaier and Graham Streets), and at more distant locations. These zones were selected to represent “source,” “intermediate,” and “control” groups, respectively. We attempted to collect wipe samples along transects representing various distances from the processor. Each location was photographed and geocoded. Methods to measure dust loading or siltation rates, which require the use of controlled and cleaned deposition surfaces or flux measurements for extended periods (given the episodic nature of FD emissions), were not utilized in favor of rapid “grab” sampling methods, described below, that are more easily applied in community-based studies.

Composite road dust and surface soil samples were collected at 20 locations (Figures 1 and 2) on 14 October, 22 November, and 21 December 2022. On the last date, several areas were resampled for confirmatory analyses. Follow-up samples included sites S24 and S25 on Dearborn Street near the metal processor and S22 on Division Street; these sites were near S03, S06, and S17, which were previously sampled. An additional sample was obtained from a nearby waste pile (S23) that was suspected as a contamination source for samples S17 and S22 based on its proximity, evidence of erosion, and levels of persistent organic contaminants (results unpublished). Each composite sample, which combined four subsamples collected within a ~2 m area, was collected from the top 5 mm of soil, sediment, or silt accumulations near curbs or sidewalks using a cleaned spatula and placed into a polyethylene bag that was labeled and sealed. Sampling avoided sticks, vegetation, and other debris. The collected weight of the field sample (before drying) was typically 150–200 g.

In addition to the soil/road dust samples, our community partners recruited participants and collected a total of 56 wipe samples at 27 residences between 17 September and 17 November 2022. Each residence was geocoded and mapped. At most residences, several samples were collected: typically a first-story vertical glass window at the front of the residence; additional samples included mostly horizontal surfaces, e.g., window sills, table tops, and ledges. Figure S3 shows pictures of typical sampling locations. Wipe samples were collected using new Kimwipes, which is a low-lint cellulose-based wiper, moistened with distilled water. For windows, the target wiping area was 100 in^2^. After collection, the wipe was folded, placed in a polybag, sealed, and labeled. For each sample, the surface type (e.g., glass, concrete, paint), area sampled, and orientation of the surface (vertical or horizontal) were recorded. The dry weight of the collected dust was estimated by drying the wipe after sampling in a desiccator, weighing to 1 mg, and subtracting the weight of a clean wipe (0.473 ± 0.009 g, N = 10).

We also obtained air quality monitoring data collected at fixed sites by the state of Michigan [17] and mobile monitoring collected by the Michigan Pollution Assessment Laboratory (MPAL) [18].

### 2.3. Sample Analysis

Soil/dust samples were dried, mixed, and sieved to <1.6 mm, and ~5 g was placed in a double open-ended X-ray fluorescence (XRF) sampling cup (1330, Chemplex, Palm City, FL, USA) covered with XRF Mylar sample film (3011, Chemplex, Palm City, FL, USA). Triplicate XRF measurements for each sample were obtained by repositioning the XRF aperture at a different section of the sample. For elemental analyses, wipes were folded multiple times to reduce their size to ~1.5 cm × 1.5 cm area, placed in a precleaned one-ton pellet press, and compacted at 1-ton pressure for 30 s to produce a dense paper pellet for analysis. The XRF sampling stage was cleaned with fresh IPA-soaked wipes between samples. Elements heavier than Al were measured with XRF using a high-definition instrument (HD Mobile 101 HDXRF Analyzer 401925–04, X-ray Operating Systems, East Greenbush, New York, NY, USA); monochromatic excitation energies of 7, 17, and 33 keV; and a spot size of 1 mm. For the wipe samples, the instrument was set to wood mode, and to glass mode for soil samples. Instrumental method detection limits (MDLs) were in the low parts per million (ppm) range for elements of interest except Cl and P, which had limits in the hundreds of ppm. The final method detection limit (MDL) was the higher of the instrumental MDL or three times the average uncertainty of the replicate measurements.

Quality assurance activities included triplicate measurements for each sample (noted above) and two metal reference checks (NIST Montana Soil Standard 2711, Gaithersburg, Maryland, USA; EC ERM 680/681K, Geel, Belgium). The triplicate measurements were averaged. Because the wipe samples included only small amounts of dust on the Kimwipe (see below), background levels on three clean Kimwipes were measured following identical protocols. These showed Br, Cd, Cl, and Sb levels comparable with those on actual wipe samples; these elements were omitted, as they primarily represented the background. In addition, Au, Ba, Ga, Hf, Hg, In, Sb, Se, Sn, and W had few if any detections above MDLs in the samples, and thus, these elements were also omitted. Repeat (follow-up) sampling at three locations showed an average relative absolute deviation (RAD) of 24% across the measured elements. Elements K and S had the greatest variation; omitting these elements dropped the RAD to 14%.

Several samples were considered to be unrepresentative based on physical characteristics or sampling location and omitted from the data analysis. These included wipe sample SWD14 labeled “truck slag found on the road,” which appeared to be an aggregation of debris, and soil sample S07 collected at >10 cm depth from a planting near the truck entrance gate.

### 2.4. Data Analysis

The small amount of mass on the wipe samples could compromise the XRF measurements. The SI describes the normalization of the samples by dividing the measurements by the reconstructed mass (RM), which was calculated as the sum of the common oxidized form of minerals based on the XRF measurements, which is a technique commonly employed for air samples [19]. This technique worked well, providing the mass fraction (expressed in ppm), which allowed for direct comparison of the different measurement types (e.g., soil and wipe samples) and reduced the measurement variability.

The road dust and soil samples were grouped into the following zones: the metal processor or source area, including road dust samples collected on Dearborn Street near the metal processor (N = 8); “comparison” road dust samples collected north, east, and south of the processor (N = 4); and “background soils,” including surface soils collected north and south of the processor (N = 6). Substrate dependencies for the wipe samples were tested by comparing window samples (N = 27), painted surfaces (N = 15), and other surface types (N = 12).

The pollution index (PI) was calculated as the ratio of the elemental concentration in the source area to the background levels measured in this study, and the enrichment ratio (ER) was calculated as the ratio of the sample concentration to the natural background levels in local soils [20]. Plots and correlation coefficients compared Detroit results to literature profiles for potentially similar sources, e.g., industrial soils and autobody shredding. Both Pearson and Spearman correlations were determined, with the latter addressing potential outliers, and statistical significance was evaluated using *p*-values.

## 3. Results

### 3.1. Composition and Spatial Distribution of Soil and Road Dust Measurements

**Figure 3.**
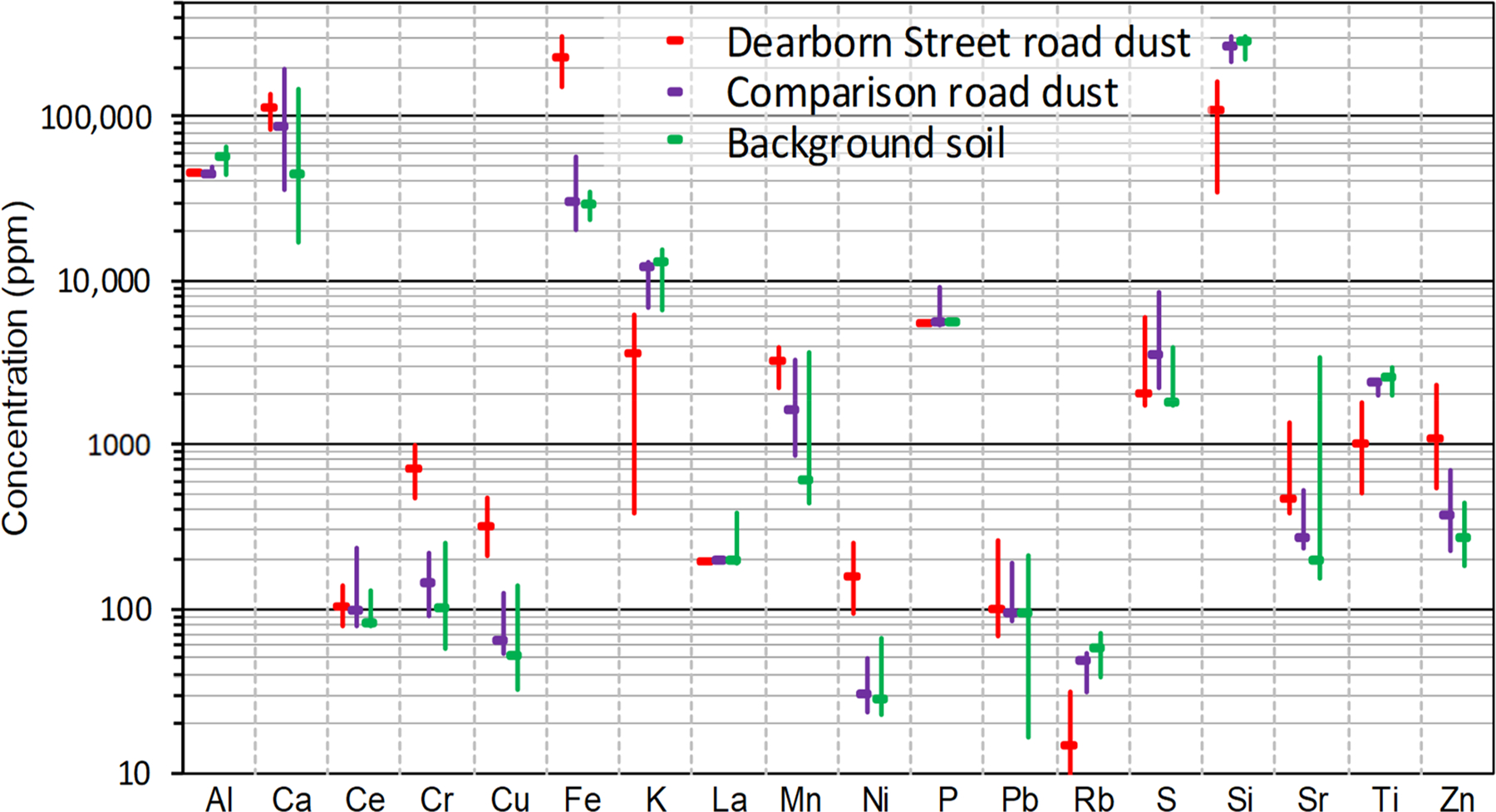
Composition of road dust/soil in three zones: Dearborn Street, comparison road dust, and surface soils (N = 8, 4, and 6, respectively). Plots show medians and ranges (minimum to maximum).

We first examined road dust and soil. Table 1 summarizes the composition of source and comparison road dust/sediment samples and background soils and lists MDLs. As shown in Figure 3, the crustal elements Ca, Si, and Al were the most abundant; some sample types had considerable P and Fe. Road dust at the processor had higher levels of Cr, Cu, Fe, Mn, Ni, Sr, and Zn levels than the comparison roads and background soils. Differences were large for Cr, Cu, Fe, and Ni, and there was no overlap between the groups. The Ce, La, and Rb levels were low (<10^−4^ ppm) or below the MDL. Measurements of these elements were not considered usable and were not considered further in the analysis.

**Figure 4.**
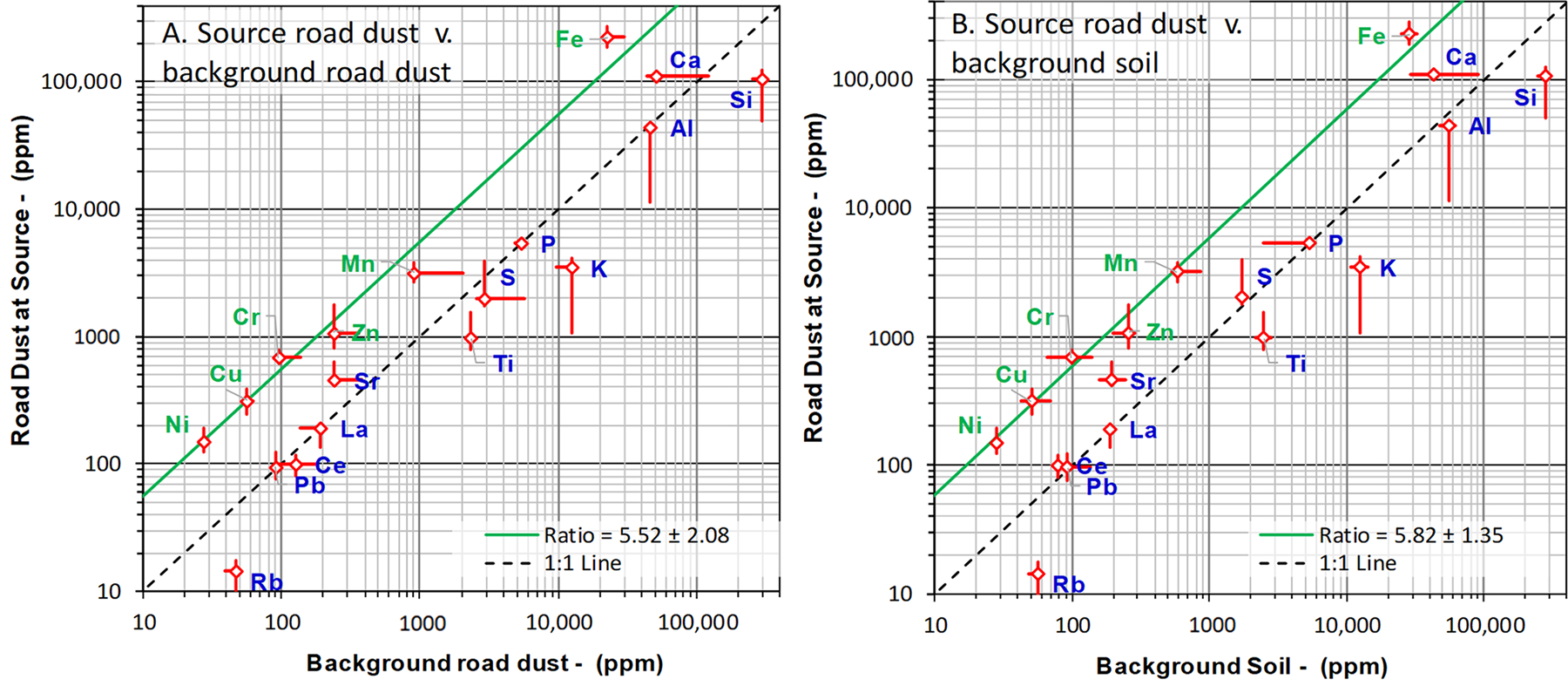
Scatterplots comparing mass fractions of road dust in the source area (N = 8) to background areas. (**A**) Source versus comparison (or background) road dust (N = 4). (**B**) Comparison with background soils (N = 6). Diamonds and red lines show medians and interquartile ranges for both axes; green line shows median ratio for elements in green; dashed black line shows 1:1 line.

Samples obtained near the metal processor were polluted compared with background soils. PIs for these samples using the median mass fraction and local background soils were 5 to 6 for Ni, Cu, Cr, Zn, Mn, and Fe (Figure 4). As discussed later in Section 4.1.1 (and shown in Table 2), levels in local background soils considerably exceeded levels measured in remote areas. With the exception of Fe, these metals had low abundances in the local background soils (mass fractions <10^−3^). The combination of a high PI and low background indicates that the metal processor was a unique or dominant source of Ni, Cu, Cr, Zn, and Mn in the road dust/sediment samples and that these metals could form a profile for this source (explored later). The similarity of Figure 4A,B (and patterns in Figure 3) suggest that road dust/sediment outside the processor area was derived mostly from surface soils.

### 3.2. Composition and Spatial Distribution of Wipe Samples

**Figure 5.**
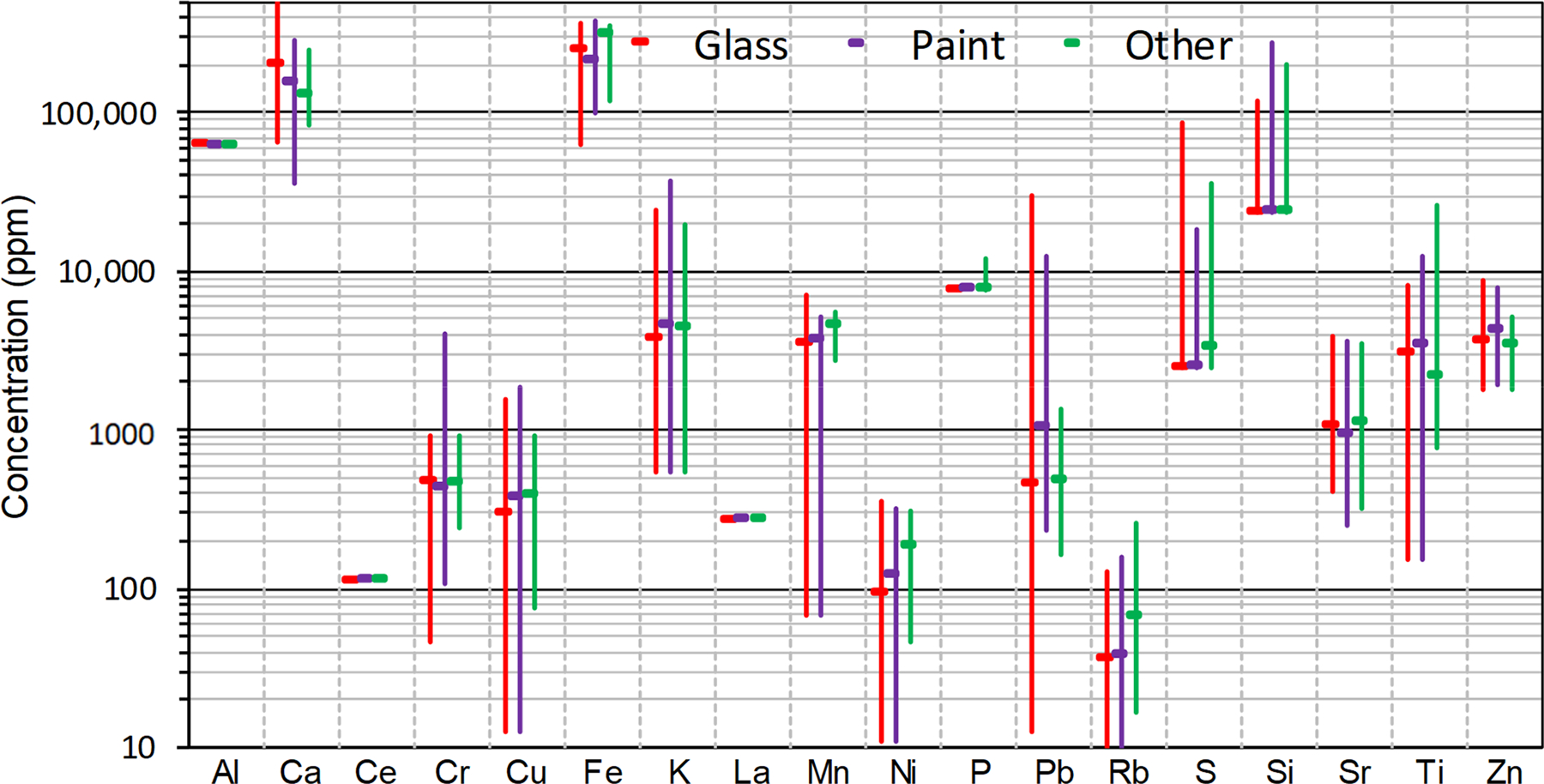
Composition of wipe samples by surface type, including glass (windows), painted, and other surfaces (N = 27, 15, and 12, respectively). Plots show median and range (minimum to maximum).

Surface dust on windows and painted and other surfaces had similar elemental mass fractions based on the t- and MW-tests (Table 1, Figure 5), in part reflecting the variability within each sample type (Table S1). Like the soil samples, the most abundant elements were Fe, Ca, and Si. Levels of Ce, La, and Rb were low (<10^−4^) or below the MDL and were not considered further. Elements potentially associated with the metal processor, i.e., Ni, Cu, Pb, Mn, and Zn, had mass fractions from ~10^−4^ to 10^−2^, and Fe was high, representing over a third of the mass in the Dearborn Street samples. One difference identified between the sample types was the higher Pb concentration on dust collected on painted surfaces, which is consistent with the use of leaded exterior paint. While the house-to-house variation was large, mass fractions of several elements collected from windows and other sample types at the 12 houses where multiple sample types were collected were moderately to highly correlated, including Ca (Spearman r = 0.67, *p* = 0.02), Cu (r = 0.59, *p* = 0.05), and Ni (r = 0.67, *p* = 0.02); other metals with moderate (but not statistically significant) correlations included Fe (r = 0.38, *p* = 0.22) and Ti (r = 0.36, *p* = 0.26). The similar composition across the three types of wipe samples, the overlapping ranges, and the correlation across sample types suggest that these data could be pooled.

**Figure 6.**
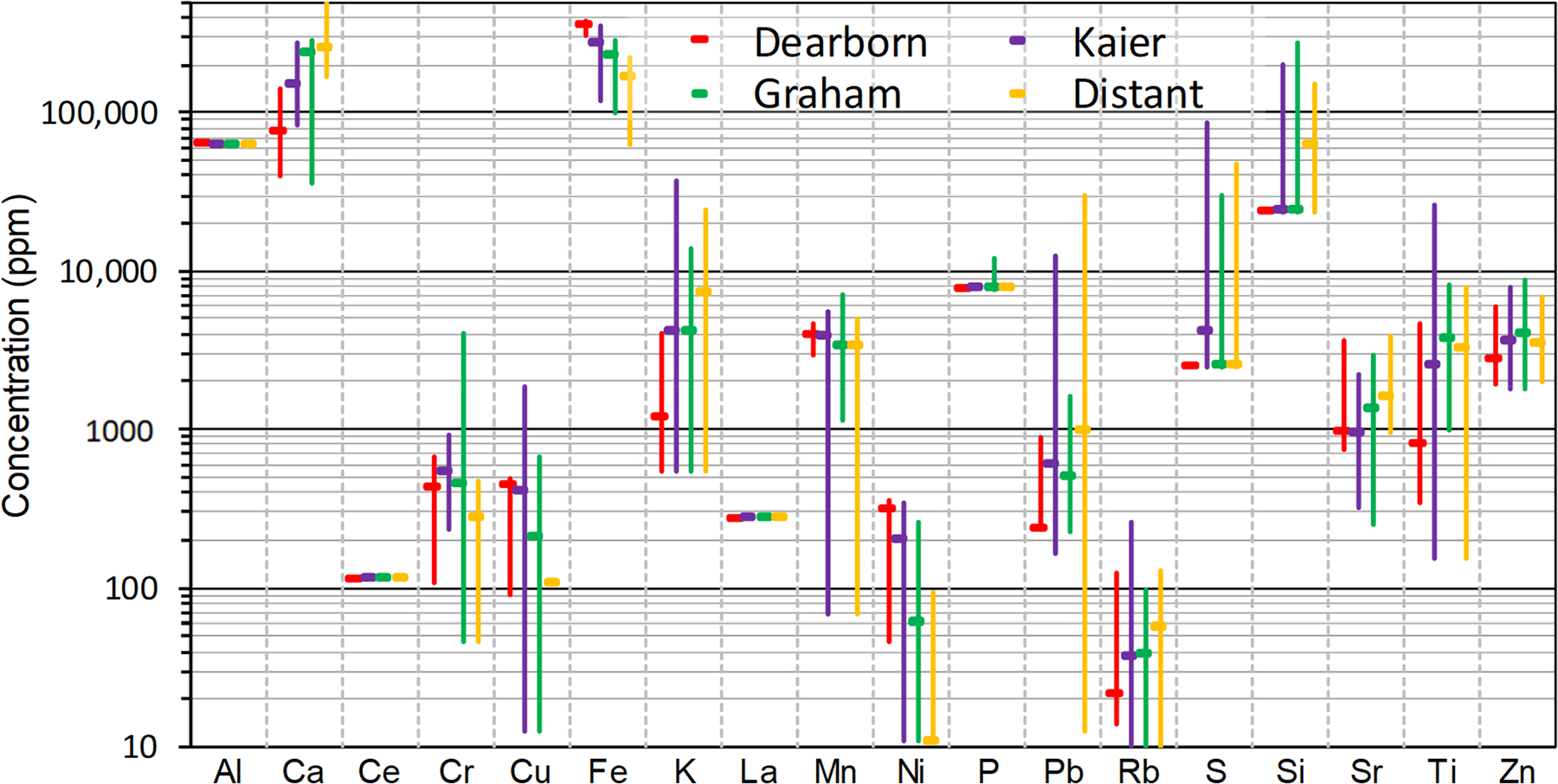
Composition of pooled wipe samples by zone (Dearborn Street near metal processor, Kaier Street just north, Graham Street just south, and distant locations; N = 5, 29, 12, and 8, respectively). Pooled samples include glass, paint, and other surfaces. Plots show median and range (minimum to maximum).

**Figure 7.**
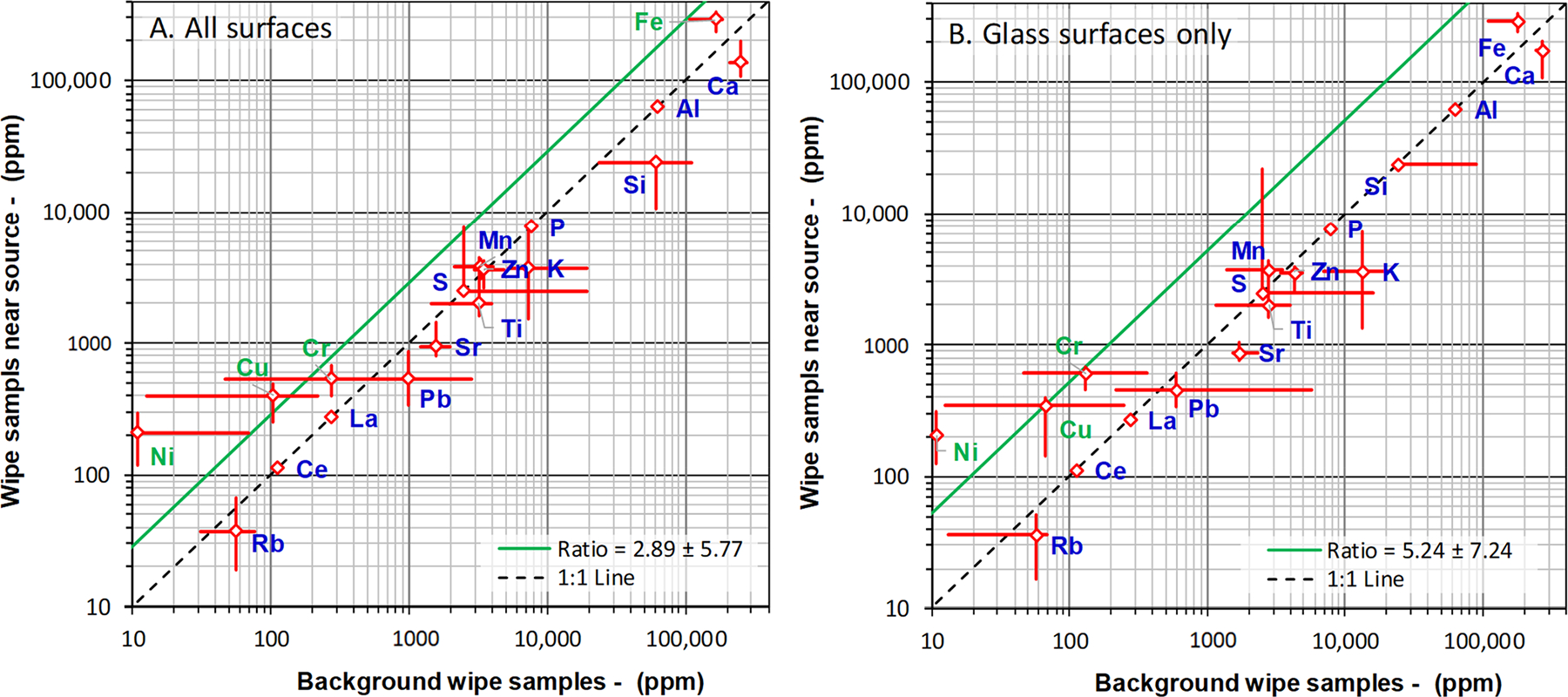
Scatterplots comparing composition of wipe samples in the source area to background. (**A**) Comparison of all surfaces (N = 34) to all background samples (N = 8). (**B**) Comparisons of only glass surfaces (N = 13) to background glass areas (N = 8). Red diamonds and red lines show median and interquartile range; green line shows median ratio for elements in green (Cu, Cr, and Fe in left plot); dashed black line shows 1:1 line.

The concentrations of several elements varied spatially. Wipe samples grouped by zone showed elevated Cu, Fe, and Ni in the Dearborn Street area near the metal processor; levels of these metals decreased in the Kaier Street area to the north, followed by lower levels in the Graham Street area to the south (Figure 6). More distant homes tended to have lower levels. Cr showed a similar trend, although the Kaier Street area did not have the highest levels. Conversely, Ca, K, Pb, and Ti showed a weak trend of increasing concentration with distance from the source. Transect plots showing mass fractions versus distance from the processor showed considerable scatter and only weak trends. Grouping the samples into 0–100, 100–200, and >200 m distances, representing near-, mid-, and far-field groups from the processor, also showed few meaningful differences. However, scatterplots contrasting the source area and background samples (using medians) showed clear differences (Figure 7A), especially for samples collected on glass surfaces (Figure 7B). PIs for wipe samples collected from glass surfaces averaged ~5 (source to background comparison), just below the PIs for the road dust. Overall, wipe sample results show that surfaces near the metal processor were elevated in several metals potentially associated with FD from the metal processor, but the differences were less pronounced than seen in the road dust and soil samples.

### 3.3. Comparison of Soil/Sediment and Wipe Samples

**Figure 8.**
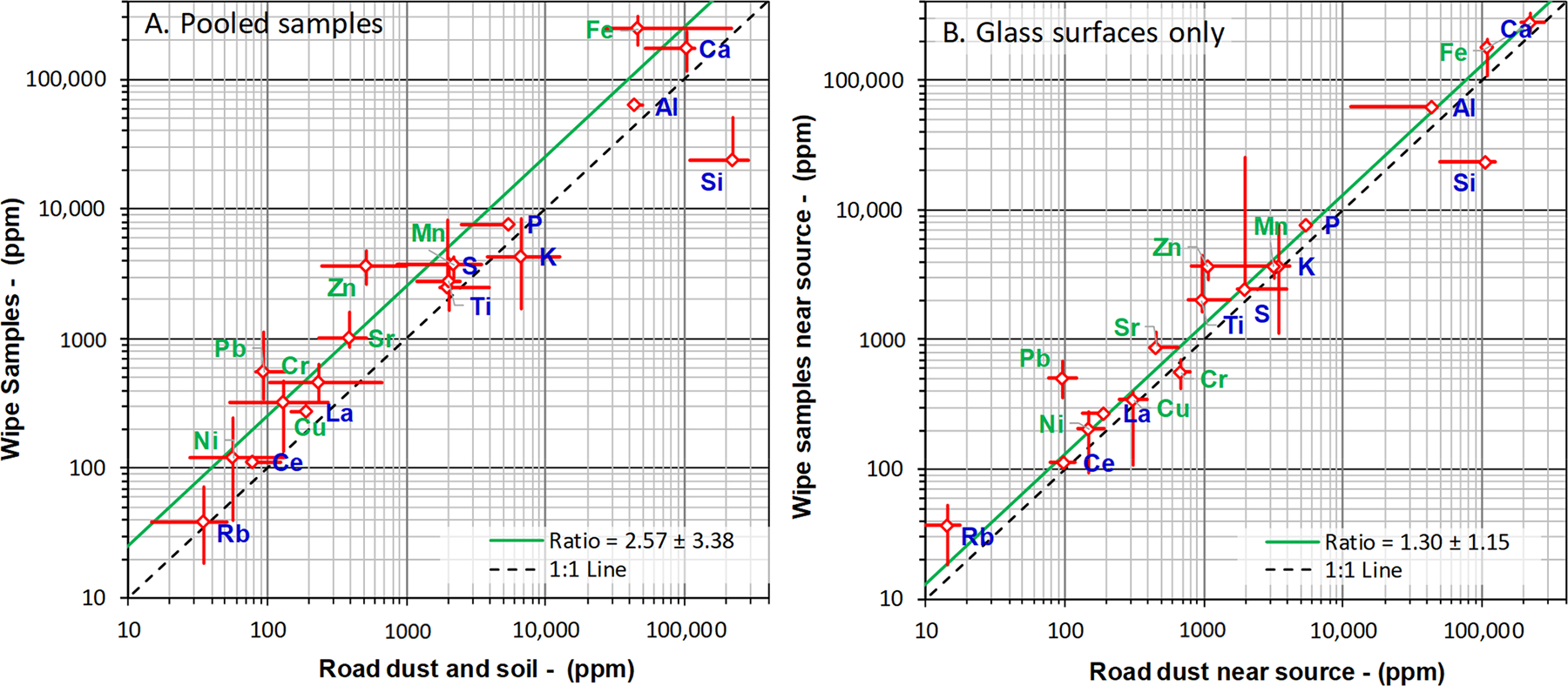
Scatterplots comparing mass fractions of wipe samples to soil/sediment. (**A**) Comparisons for all surfaces (N = 54) with all soil/sediment samples (N = 18). (**B**) Comparisons restricted to source area for wipe samples from glass surfaces (N = 12) with soil/sediment samples (N = 8). Red diamonds and red lines show median and interquartile range; green line shows median ratio for elements in green; dashed black line shows 1:1 line.

The road dust, soil, and wipe sample results are compared in Figure 8. Across all locations and sample types, wipe samples had higher metal concentrations, typically by a factor of 2.6 (median ratio; green line in Figure 8A); this factor dropped to 1.3 by restricting the comparison to wipes collected on glass surfaces near the metal processor (Figure 8B), showing more comparable results. The levels of all metals (Cr, Cu, Ni, Mn, Fe, Zn) in road dust collected near the processor considerably exceeded (by 5–6 times) the levels in background soils (Figure 4). For the wipe samples, only Cu, Cr, and Ni showed this trend (Figure 7). Overall, wipe samples had higher metal concentrations, increased variability, and attenuated differences between source and background areas compared with road dust/soil samples.

The elevated metal levels and diminished contrast between source and background areas in the wipe samples could have resulted from several factors. First, road dust and wipe samples were not always obtained at the same location (e.g., few wipe samples were obtained on Dearborn Street due to a lack of homes). Second, wipe samples represent an accumulation of primarily small particles (especially on vertical sources) that can arise from both local and distant sources, e.g., fly ash that contains relatively high levels of refractory metals. In Detroit, about half of PM_2.5_ is sulfate and nitrate aerosol accompanied by trace metals from fuel combustion at distant sources, including coal-fired facilities [23], and only small differences in concentrations across the study area were expected for such aerosols. Third, normalization may introduce bias, particularly for the wipe samples, since the mass associated with carbonaceous aerosols, which represent ~20% of PM_2.5_ in Detroit, was not measured; this component will be smaller for the road dust/sediment samples.

The greater variability in the wipe samples resulted from several factors. First, wipes collected relatively little mass, resulting in less accurate XRF determinations. Second, road dust/sediment/soil collection used composite samples, which accumulated contaminants over an unknown but extended period. The multiple subsamples constituting each composite sample increased the spatial representativeness, and the long accumulation period provided temporal “averaging” that also increased the representativeness. In contrast, the wipe samples collected surface dust over a small area with an unknown and potentially short accumulation time, although most areas were visually “dirty.” Third, wipe samples could include residues from the substrate, as suggested for Pb from paint. Additional variability could result from the heterogeneous nature of the wipe samples, the skewed distribution of concentrations, and the limited sample size.

## 4. Discussion

### 4.1. Comparison with Literature

#### 4.1.1. Soil Surveys

Several soil and road dust surveys in Detroit were conducted recently, as summarized in Table 2. In 2019–2020, 47 road dust and 4 soil samples were collected in the Detroit area and analyzed using ICP-MS, with the findings that the Cr, Ba, Zn, Pb, and Se levels were moderately to highly elevated, and Ba, Cr, and Zn were stated to exceed the “EPA limit” based on a non-cancer screening level with a hazard index of 0.1 (Table 3 in Yang et al., 2023 [23]). Howard et al. [21] sampled road dust and sediment at 23 Detroit locations, which were analyzed using XRF and other techniques; the study noted the widespread distribution of microspheres in residential soils that reflects fly ash deposition, a legacy of residential coal-burning (from ~1850 to 1936), and intensive industrialization (~1890 to the present). Sources of several metals were discussed, e.g., Pb was attributed to leaded gasoline emissions, exterior house and road paint, and building demolition. In a third and related study, microartifacts and elemental concentrations in 35 soil samples obtained from demolished and intact residential and industrial areas in Detroit showed distinctive elemental compositions for coal, iron slag, and fly ash particles (which were differentiated using electron microscopy); microparticles at an industrial site had high levels of Fe, Cu, Ti, and Zn [24]. Murray et al. [22] analyzed 3786 soil samples from an urbanized area of southeast Michigan (including Detroit) and found elevated levels of Sb, As, Ba, Be, Cd, Cr, Cu, Pb, Hg, Ni, Se, Ag, Tl, and Zn in surface soils in industrial areas; Pb was often elevated near older homes. These Detroit area studies show widespread contamination in urban areas and considerable variation due to local conditions.

Table 2 also lists natural background levels in Michigan soils at sites believed to be unaffected by anthropogenic releases [20]. Background levels depend on the soil type (e.g., sand and clay topsoil), parent material, geology, and glacial history. The table shows the mean (or median) levels by soil type for the state and for southeast Michigan (using the Huron–Erie lobe encompassing Detroit), and the statewide upper range (97.5 percentile), which is sometimes used to delineate contamination for site remediation purposes. A heatmap (red is highest) illustrates the concentration gradient for key elements across the data sets.

Compared with the literature data, our road dust/sediment samples were significantly elevated in Cr, Cu, Fe, Mn, Ni, Pb, and Zn over background levels, especially samples from the metal processor area. Our “urban background” soils were elevated in Cr, Pb, and Zn relative to the natural background, but the levels of Cu, Fe, Mn, and Ni were similar.

#### 4.1.2. Air Quality Monitoring

Short-term air quality measurements were obtained at the processor site by our mobile air quality monitoring platform (MPAL) during surveys conducted in portions of Detroit since 2019 [18]. The MPAL was parked on Kaier Street opposite the metal processor on two occasions after visiting a large construction site. Unusually elevated Fe levels were detected on both occasions: 30 min samples showed Fe concentrations from 0.6 to 5.4 μg/m^3^ (using XRF), while PM_10_ levels ranged from 30 to 82 μg/m^3^ (N = 3, 30 min averages, 11:00–12:00 on 16 August 2019, 12:30–13:00 on 14 February 2020). The SI describes these and ancillary measurements (Tables S2 and S3, Figure S5). While we cannot definitely assign the source of Fe and PM_10_ and the results are not representative given the monitoring’s short duration, these results show that PM_10_ and Fe were elevated in this area of Detroit.

Long-term records of metal concentrations are available at seven permanent sites within 4.1 km of the metal processor. Three particle size fractions were monitored: TSP (seven sites), PM_10_ (one site), and PM_2.5_ (two sites). The Dearborn site (1.8 km NNE of the processor) was the location used for measuring the three size fractions. The most recent complete year of data available, namely, 2021, is summarized in Table S1. For TSP, annual average levels of As, Cd, Mn, Ni, and Pb spanned a two-fold range across the seven sites; the Trinity site nearest the study site (0.46 km NE) had the highest levels of As and Ni. Dearborn showed much higher (5–20 times) levels of Fe and Mn in larger PM size fractions (TSP, PM_10_) than the smaller fraction (PM_2.5_), while As, Cd, Cu, Ni, Pb, and Zn levels were similar. This was consistent with larger PM being dominated by crustal components, while smaller PM reflected trace and refractory metals associated with combustion, mobile sources, and some industrial activity.

Despite the short duration of monitoring, the MPAL data show that FD at the processor site was elevated in Fe; the fixed sites were too distant to show these impacts, e.g., TSP data did not show the enrichment of Cu and Zn compared with Fe, Mn, Ni, and Cr, as seen in local road dust/sediment (Figure S4). These results are unsurprising given that the metal processor is only one of the numerous emission sources in the area and the “hyperlocal” impact of the metal processor, which results from the very short travel distances for the large particles in FD [26]. Notably, a recent source apportionment at three Detroit monitoring sites identified both ferrous and non-ferrous metal sources that contributed to the PM_2.5_ levels, particularly at Dearborn, and profiles for these sources were distinguished using the abundance of Cu, Fe, and other metals [23]. While these sources contributed only a modest fraction (<6%) of PM_2.5_, the present study suggests considerably larger contributions for PM_10_ and TSP near FD sources, like scrap metal facilities.

#### 4.1.3. Chemical Profiles

**Figure 9.**
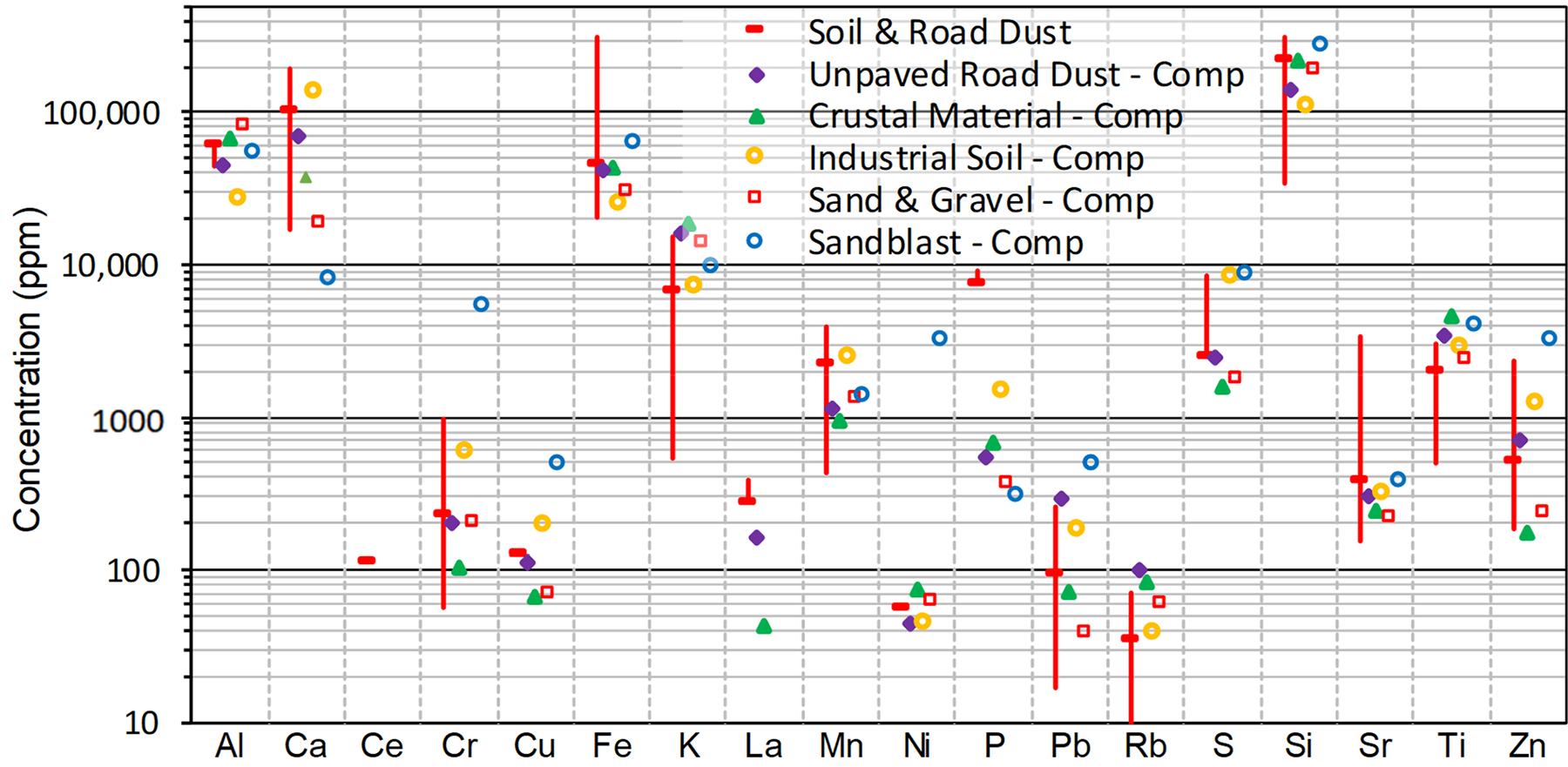
Comparison between soil and road dust samples in Detroit and five profiles from the EPA SPECIATE dataset (“Comp” denotes composite profiles). Medians and range (minimum to maximum) are shown for the Detroit road dust and soil samples (N = 18).

Chemical profiles developed for a variety of applications have been compiled in the large SPECIATE dataset [25]. Five “recommended” profiles from this dataset that were possibly similar to those measured in Detroit were selected: unpaved road dust, crustal material, industrial soil, sand and gravel, and sandblast dust. Each is a composite profile (derived from multiple sources). These profiles are diverse (Figure 9): the crustal material profile had low levels of trace metals (e.g., Cr, Cu, Pb, and Zn), the sandblast profile had the highest levels, common crustal elements (Al, Ca, and Fe) had a large (~fivefold) range across the five profiles, and Si was more consistent (twofold range). Several profiles resembled the Detroit data: unpaved road dust had similar Cr, Cu, Ni, S, and Zn levels, and industrial soil had similar Al, K, Mn, and P levels (although the Detroit samples had higher P and lower Ti). The correlation analysis shows these and several additional associations with Detroit road dust (Table 3): the industrial soil profile had the strongest (Spearman) correlation with road dust in both the source area (R = 0.70, *p* < 0.01) and other Detroit areas (R = 0.69, *p* < 0.01); other statistically significant correlations (*p* < 0.05) occurred for the literature profiles for unpaved road dust, sand and gravel, and tire dust. In contrast, the literature profiles had a near-low correlation and a statistically insignificant correlation with Detroit background soils. These findings suggest that the Detroit road dust was due to multiple sources since even the highest correlation explained less than half of the variation and the noted sources (e.g., industrial soil and tire dust) were consistent with site activities. However, results may also reflect differences attributable to factors noted earlier, e.g., spatial variation, different sample types, and analytical differences. We also note that the SPECIATE database is intended to represent airborne (or resuspended) PM, but not surface soil or dust. Despite such differences, the Detroit data showed modest agreement with unpaved road dust and industrial soil profiles.

“Enrichment ratios” (ERs) comparing measurements to the crustal average (using SPECIATE profile 91169) are listed in Table 4 for the Detroit road dust and soil profiles, the four SPECIATE profiles discussed earlier (the fifth is the denominator in the ER), and several other possibly relevant profiles in the SPECIATE database. The elevated Cu in the road dust near the metal processor was roughly matched by the sandblast, electric arc furnace, and autobody shredding profiles, but few other metals in these profiles were comparable. The elevated Fe in road dust near the processor was matched by the autobody shredding profile, as were Cr, Cd, Mn, Ni, and Zn levels, but this profile’s Ca, Pb, S, and Sr levels exceeded levels in the road dust. Road dust near the processor and industrial soil had similar ERs for Ca, Cr, Mn, P, Pb, S, and Zn, but the Detroit road dust also was enriched in Fe and Ni. Overall, the literature profiles did not match the metal processor dust. In contrast, the Detroit background soil profile had ERs between 0.5 and 1.5, showing a good match to the literature crustal profile (with the exception of P with ER = 5.1, but our P measurements were unreliable given the high MDL).

The high Fe concentration in the road dust/sediment collected near the metal processor was the most striking feature of the profile. This was consistent with mill scale, which is a type of iron oxide formed on the surface of steel during the high-temperature and high-pressure hot-rolling process. Other sources of high Fe levels in the iron and steel production process have been documented, e.g., sinter cake discharge zone, blast furnace, casting, desulfurization, and slag processing [27], but Fe in FD in a community setting appears to be a novel finding and possible health concern, as described below.

### 4.2. Health Risks

Contaminants in FD accumulate on roads, soils, and other surfaces and can expose people directly due to ingestion, dermal absorption, and inhalation, and can cause indirect exposure due to the consumption of crops, animal products, or water that has become contaminated [28]. The potential for health risks is generally assessed by comparing environmental measurements to screening levels that represent concentrations believed to present a minimal and negligible likelihood of an adverse health outcome. Typically, screening levels address cancer risks using a concentration representing a one in a million chance of cancer, and for non-cancer outcomes, using a concentration expressed as the no or lowest observed adverse effects level (NOAEL, LOAEL) for the most susceptible and exposed subgroup, which is often children since they consume more soil per body weight than adults. The U.S. EPA lists screening levels for soil in residential settings [29]; some jurisdictions adjust levels for commercial and industrial land uses where prolonged exposure is unlikely, and some states specify alternative limits for direct contact with soils. The current screening levels are shown in Table 2.

Based on zone medians, the road dust/sediment samples collected near the slag processor had Fe levels (224,000 ppm) that exceeded the U.S. EPA non-cancer screening level (55,000 ppm) by fourfold and the Michigan contact guideline (160,000 ppm) by 1.4-fold. No other element exceeded the screening levels. However, for additional conservativeness, sometimes an additional safety factor of 10 (using a hazard index of 0.1) is incorporated into non-cancer screening levels, which would additionally flag Al, Cu, Ni, and Pb. The Fe screening level is derived from the Provisional Peer Reviewed Toxicity Values, which specifies a LOAEL of 1 mg/kg-day based on adverse but reversible gastrointestinal effects associated with dietary exposure to iron supplements for 1 month and an uncertainty factor of 1.5 [6].

We did not identify other community settings in the literature where Fe in soils or in FD exceeded health-based guidelines. Relatively few studies examining urban soils even report Fe. More commonly, toxicity in urban soils and dust has been associated with other metals, e.g., Pb, Zn, Cu, Cr, and Ni in Europe [7] and Cr, Ni, Cu, Pb, Zn, As, Hg, and Cd in China [8].

Our analysis showed localized impacts from the metal processor. From storage piles themselves, modeling suggests that maximum impacts will occur within 75 m [26]. Truck track-out may extend this distance, depending on local conditions (e.g., loading, moisture, housekeeping). As noted, iron and steel making are well-known sources of PM and FD, even from enclosed storage yards [30]. This study suggests the significance of FD from scrap collection and processing.

### 4.3. Improving FD Assessment and Management

#### 4.3.1. FD Emissions Inventories

Urban and industrial areas can contain many sources of PM and FD, some of which are compiled in emissions inventories. However, inventories have significant limitations for FD: generally, only county-wide estimates are available; many sources are excluded; PM greater than 10 μm is excluded; and compositional information is absent. The following explores several of these issues.

Due to its manufacturing legacy, Detroit has considerable industry. The 2020 U.S. EPA National Emissions Inventory (NEI) lists 165 point sources in Wayne Country (encompassing Detroit) with PM_10_ emissions totaling 1697 tons/year [3]. The largest sources include Rouge Industrial Complex now the Cleveland Cliffs Dearborn Works (sheet steel production with blast furnaces, waste oxides reclamation facility, continuous casters, hot strip and cold mill operations); U.S. Steel (Detroit and nearby Ecorse: integrated steel mill with hot strip mill, scale pit, iron- and cokemaking, basic oxygen process, pickle and electro-galvanizing lines, cold mill, annealing furnace, boiler house); EES Coke (Zug Island: coke and by-product production using coke oven, coke by-product recovery plant, boiler houses); Marathon Ashland Petroleum Refinery (140,000 bbl/day processor of sweet and heavy sour crudes); Ford Motor Company Rouge Complex (Dearborn: vehicle assembly, engine and fuel tank production, stamping plant, frame manufacturing, coating plant); Dearborn Industrial Generation (boilers and flares combusting natural gas and blast furnace gas); Cadillac Asphalt Products Corporation (hot mix asphalt, up to 940,000 tons/year); U.S. Gypsum (River Rouge: wallboard and cement board maker, grinding and drying operations); Carmeuse Lime (lime production: rotary kilns, dust tank); Detroit Wastewater Treatment Plant (sludge incinerators, ash transported for land disposal); St. Mary’s Cement (grinding mills); Edward C. Levy Co Plant 1 (rock and slag processor, hauling on paved and unpaved roads; Darling International, Inc. (Melvindale: rendering operation with boilers, grease and oils processing); and several DTE Power Plants (Detroit and River Rouge: gas and coal fired boilers). Facility emissions of PM_10_ are dwarfed by area and nonpoint emissions, which total 13,409 tons/year in the county and are dominated by unpaved road dust, construction dust, paved road dust, and industrial processes/mining (3251, 3123, 1410, and 1136 tons/year, respectively).

Because site-specific information on the composition of emissions is unavailable, assessments depend on generic information in libraries, as noted earlier [25]. Our data suggest that existing profiles may not adequately characterize sources such as the scrap metal processor investigated. Recommendations to improve emissions inventories [31] were implemented in recent decades and improved emission estimates can now be derived for sources, such as paved and unpaved roads and construction [32]. It is appropriate to identify and separate the types of FD sources, e.g., industrial facilities handling toxic materials, especially those located near residential areas. As shown below, the inventory does not capture most metal sources potentially causing hyperlocal impacts.

#### 4.3.2. Metal Processers in the Study Area

The existing emissions inventories excluded the metal processor in this study and most other metal storage, recycling, and processing facilities. As a preliminary investigation to identify potential FD sources associated with metal processing, we automated searches using Google Maps and Yellow Pages (http://www.yellowpagecity.com, accessed on 9 July 2023) with keywords including Detroit and alloy, aluminum castings, annealing, coatings, iron and iron work, metal fabricators, metal cutting machines, metal finishers, metal heat treating, metal powder fabricating, metal products, metal rolling and forming, metal roofing and siding, metal slit and shear, metal stamping, non-ferrous, scrap, steel products and processing, and welding. Sales and distribution facilities were eliminated. The remaining listings were web-searched to identify activities, geocoded, and grouped into descriptive categories.

**Figure 10.**
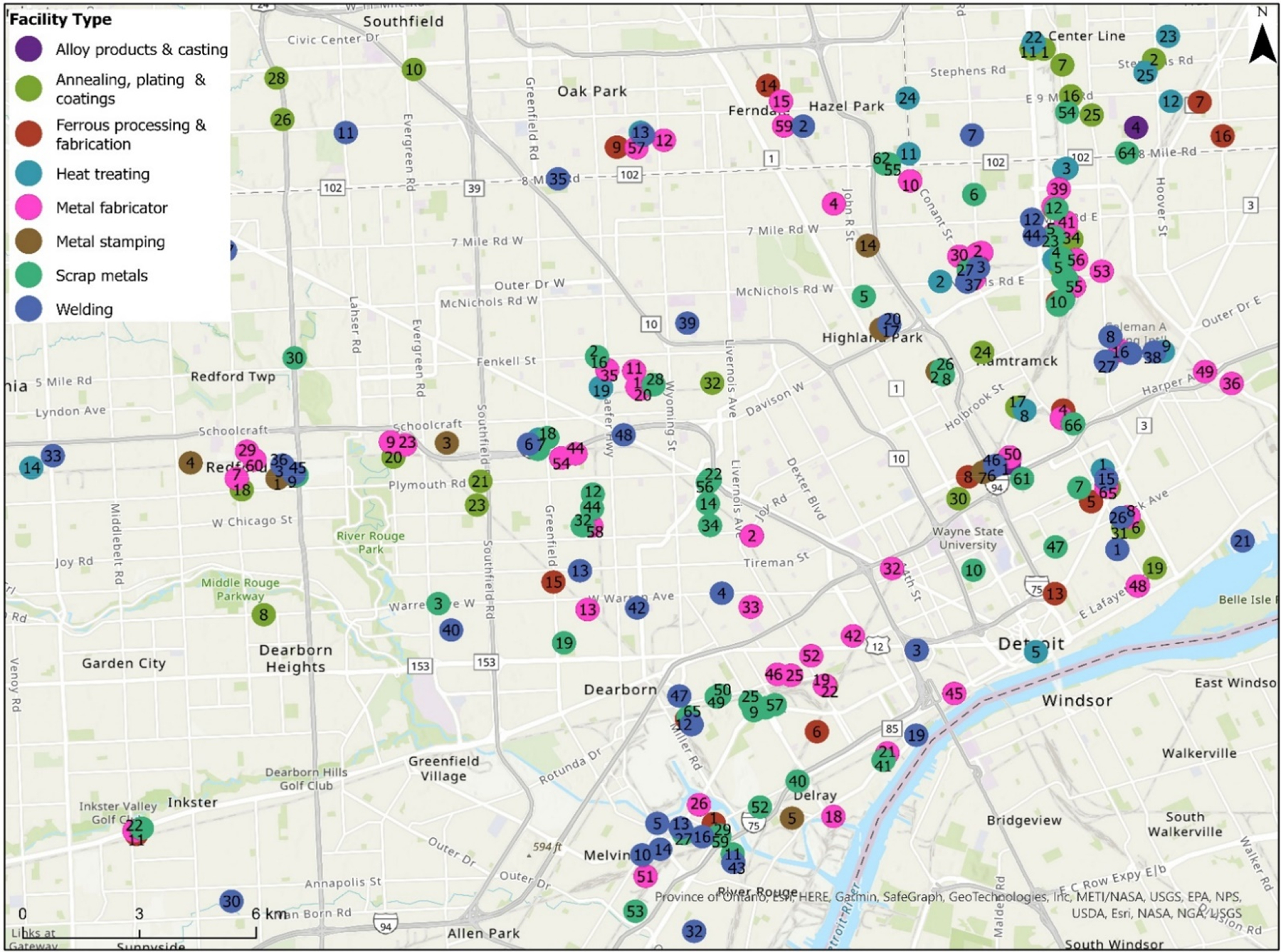
Map showing locations of metal processing facilities, coded by facility type, in the study area. Specific facilities identified in Table S4.

The number of metal handling facilities in Detroit is striking. Figure 10 maps 271 facilities in Detroit, which include 67 scrap metal facilities; 60 metal fabricators; 48 welding firms; 34 annealing, plating, or coating operations; 28 heat treating facilities; 18 ferrous processors/fabricators; 10 metal stamping plants; and 6 alloy casters/producers (listed in Table S4). Many facilities are clustered, but others are dispersed across the area. The processor examined in this study (location 52 in Figure 10) is not the largest (by property area) nor located in the largest industrial cluster. While some FD emissions from these facilities may be captured in the county-aggregated non-point PM_10_ NEI estimates, this does not allow for the assessment of localized impacts, nor are these sources identified as “industrial soils” or other source type (e.g., using the literature profiles discussed in Section 4.1.3). Potentially, small facilities might be considered as non-point or area sources and larger facilities as point (facility) sources.

The impact of FD emissions from metal processors is suggested by several factors. First, pollution indices for metals in surface soils and dusts were elevated near the case study metal processor. While this might result from contaminated run-off and not airborne fugitive dust, this was clearly not the case here. Second, air quality monitoring in Detroit (Section 4.1.2) shows that the levels of airborne metals exceeded “background” levels, and the receptor modeling analysis (cited in the same section) found that both ferrous- and non-ferrous metal sources contributed to PM_2.5_, both suggesting local sources. Third, while steel and coke production in Detroit is captured in the NEI, the compositional profile of these sources did not match the dust found near the processor (Section 4.1.3).

We recognize that our investigation of metal handling facilities is preliminary, and we made no attempt to estimate the facility size, activities, or emissions. Although retail sales and distribution facilities were excluded, some facilities may be misclassified and have no emissions. Site inspections and additional analyses are needed to confirm the potential to emit and to estimate emissions. While the number and size of metal processing facilities in Detroit may differ from other cities, the sheer number of scrap and metal processing facilities suggests that the FD from metal processors may be widespread. Potential health and nuisance concerns associated with FD can be reduced by reducing source emissions, e.g., paving work areas, enclosing operations, capturing emissions, truck tire washing, and restricting pile size and drop heights; by enhancing the oversight of such controls to ensure compliance, e.g., using regular inspections, assessment, and FD monitoring; and by separating FD sources from populations, e.g., using (vegetated) buffers and zoning.

### 4.4. FD Monitoring and Community-Based Research

FD can be monitored using several approaches. U.S. EPA methods 9 and 22 [33,34] use visual observations to identify emission sources and emission events. These methods are qualitative, limited to a portion of a source area, and may not be applicable in community settings given the needs for site access, appropriate sight angles, lighting, and data interpretation, although the methods might be adapted and automated using video, timelapse photography, image recognition software, and surveillance cameras [35]. Fence line monitoring is also used, most commonly for PM_10_ or TSP, which can detect FD emissions if a monitoring site is downwind. The availability of low-cost sensors increases this method’s feasibility, as demonstrated in several applications [36]. Ambient monitoring data are usually referenced to ambient guidelines, standards, and risk-based levels. A third approach uses deposition or dustfall monitoring, which was more common in the early-to-mid-20th century when bucket-like collectors were used; values for the study area (Detroit/Windsor) were distressingly high, ranging from 31 to 982 tons/mile^2^-month in the 1950s [37]. More recent deposition and dustfall techniques use dust traps, sticky or other surrogate surfaces, bucket-type sampling, and vacuum/suction sampling [38]. These methods have constraints, e.g., siting criteria and the need for extended periods of dry weather. Fourth, as tested here, wipe samples can be obtained from many surfaces, e.g., windows and plants. The mass collected depends on the time the substrate has been collecting material, its type, orientation, and location. Dustfall/deposition may be quantified if the substrate is cleaned before sampling and its area and mass are determined. Finally, as used here, “grab” samples of road dust and soils can be analyzed to show the presence of toxics, but not dustfall or deposition rates.

In comparing sampling approaches, we found that elemental compositions measured from road dust samples and wipe samples were similar, but the former was preferable due to better accuracy, representativeness, coverage (not restricted to accessible glass surfaces), and convenience (quick to sample and without permissions on public land). This conclusion may not apply to all situations, e.g., wipe samples collected from pre-cleaned substrates can quantify deposition rates (which is not feasible for road dust or soil samples), and in cases, the amount of road dust might be limited (e.g., requiring vacuuming), although this is unlikely where FD is problematic. Potentially, relatively few samples may be sufficient to track contamination and distinguish specific sources.

The collection of road dust, soil, and wipe samples are amenable to community-led or community-based research given the ease of sampling, the low cost of XRF analyses (~USD 10/sample), and the simple and easy-to-understand procedures, which also promote inclusion and community involvement [39]. In cases, citizen groups have used these techniques to collect and analyze thousands of samples [11,40]; this scale is infeasible without volunteers, and it allows both zone and grid-based sampling approaches [41]. Other suggestions include focusing on surficial soils, sampling a variety of urban land uses, and considering emerging contaminants [5].

## 5. Conclusions

Road dust and sediment in an environmental justice community near a scrap metal processor were contaminated with metals, especially Fe (224,000 ppm), and Ni, Cu, Pb, Mn, and Zn levels (100 to 10,000 ppm) also were well over background levels. The Fe level exceeded screening levels, indicating a potential health risk. Wipe samples showed similar profiles and indicated FD migration and deposition to nearby residential areas. Road dust near the processor differed from literature profiles, suggesting the need for local or site-specific compositional information. We show the utility of using low-cost community sampling techniques and XRF analyses, the advantages of using road dust/sediment and soil samples compared with wipe samples, which were highly variable, and the benefits of normalizing analytical measurements using reconstructed mass, which aided data interpretation and reduced variability.

The number of scrap metal and metal handling facilities in the Detroit area is striking. Emissions inventories and ambient monitoring, including PM_2.5_ and PM_10_, do not reflect possible or likely impacts associated with FD from this industry sector, which may be localized yet still objectionable given the level and composition of FD. In these cases, state permits and local ordinances can utilize controls to limit FD emissions and truck track-out; however, the coverage, stringency, and enforcement of FD controls are incomplete and appear inadequate. Collectively, these concerns suggest that environmental issues associated with metal processing are widespread. We show that conventional fixed-site monitoring of metals and PM_10_ does not capture concerns arising from FD and that “hyperlocal” studies are needed to understand impacts from specific facilities that generate FD. At the studied facility, and potentially at many other metal processors and industrial sites, the potential for FD emissions should be evaluated by inspection, assessment, and monitoring, and controls should be strengthened to limit or eliminate nuisance and contamination impacts.

## Supplementary Material

Supplementary Material

**Supplementary Materials:** The following supporting information can be downloaded at: https://www.mdpi.com/article/10.3390/environments10120223/s1, These include discussions of the normalization of XRF measurements and ambient PM measurements by the mobile facility. Also included are the following figures and tables: Figure S1. 1949 aerial view of processor. Figure S2. Photos showing aerial views of facility. Figure S3. Photos showing examples of wipe sampling locations. Figure S4. Comparison of ambient concentrations of available metals in TSP at the Dearborn monitoring site (annual average for 2021) to soil/dust mass fractions for road dust in the source area, road dust in other areas, and background soils. Figure S5. 1 min PM10 concentration trends measured using Horiba and OPC over the entire sampling visits to Detroit on 16 August 2019 and 14 February 2020. Table S1. Annual average concentrations of selected elements (ng/m^3^) and PM (μg/m^3^) in ambient air in three size fractions at local monitoring sites for 2021. Table S2. PM_10_, selected element, and other measurements by MPAL during the three 30 min periods. Table S3. PM_10_, Fe, and Ca concentrations measured using Horiba PX-375 over the entire sampling visits to Detroit on 16 August 2019 and 14 February 2020. Table S4. List of metal-processing firms identified in Detroit.

## Figures and Tables

**Table 1.** Median mass concentrations (ppm) in wipe samples, road dust, and soil samples grouped by location. N denotes sample size in group. MDL is method detection limit. Excludes elements with only non-detects.

	Road Dust and Soil Samples				Wipe Samples				
Element	Street Dust Source Area	Street Dust Comparison	Soil	MDL	Dearborn	Kaier	Graham	Distant	MDL
Al	44,122	76,640	99,790	44,122	62,639	62,639	62,639	62,639	62,639
Ca	111,631	88,165	44,714	1641	76,804	153,028	237,393	252,631	2330
Ce	179	174	118	79	113	113	113	113	113
Cr	718	171	131	33	475	583	497	320	46
Cu	323	72	60	9	445	411	216	116	13
Fe	224,374	29,940	29,043	492	355,447	272,095	231,080	166,882	698
K	3865	12,121	12,961	380	1712	4681	4648	7786	539
La	303	251	258	192	272	272	272	272	272
Mn	3249	1620	631	49	3958	3855	3458	3369	69
Ni	158	37	36	8	315	211	70	11	11
P	5407	10,321	7247	5407	7676	7676	7676	7676	7676
Pb	104	102	102	9	249	600	516	992	12
Rb	16	49	58	2	24	40	41	59	3
S	3747	5172	3015	1747	2480	6626	2480	2480	2480
Si	122,379	282,903	300,909	16,721	23,739	23,739	23,739	85,043	23,739
Sr	462	265	199	5	965	940	1339	1582	7
Ti	1080	2468	2599	109	944	2679	3868	3369	154
Zn	1079	380	275	15	2753	3639	3973	3506	21
N	8	4	6	-	5	29	12	8	-

**Table 2.** Comparison of road dust, sediments, and soils in Detroit and Michigan. Concentrations in ppm. Howard data from their Table 2. Heat maps display trends for the key elements in this study. “-” denotes not available.

	Detroit Road Dust/Sediment				Detroit Urban Soils				Michigan Natural Background								US EPA	
	Literature		This Study		Literature			This Study	Top Soil		Sand		Clay		Recommended		Screening Levels	
Element	Howard et al., 2019 [21]	Denny et al., 2022 [12]	Street Dust-Source Area	Street Dust-Other	Howard et al., 2019 [21]	Murray et al., 2004 [22]	Denny et al., 2022 [12]	Soil	SE Michigan	Statewide	SE Michigan	Statewide	SE Michigan	Statewide	Default Background	Upper Range Background	Cancer	NonCancer
Ag	-	0.5	-	-	-	1.5	0.2	-	<0.25	<0.25	<0.20	<0.18	<0.50	<0.25	1.0	1.4	-	390
Al	-	-	44,122	76,640	-	-	-	99,790	4554	2141	3024	2404	7445	7318	6900	16,014	-	77,000
As	13	8	-	-	26	7	7	-	6	2	4	2	7	5	6	23	0.8	35
Ba	-	343	-	-	-	122	381	-	40	24	28	13	64	48	75	172	-	15,000
Be	-	0	-	-	-	-	-	-	<0.20	<0.30	<0.20	<0.20	0	0	-	1.0	1600	160
Cd	9.2	1.1	-	-	3.4	3.7	0.7	-	<2.0	<2.0	<0.24	<0.20	<1.1	<0.66	1.2	2.0	2100	7
Co	-	-	-	-	-	-	-	-	<5.0	<5.0	7	4	10	9	7	27	420	23
Cr	136	207	718	171	212	58	99	131	13	6	4	3	17	14	18	56	-	0
Cu	-	96	323	72	-	78	48	60	10	6	7	4	14	12	32	51	-	3100
Fe	-	-	224,374	29,940	-	-	-	29,043	9476	4065	5863	4351	18,110	14,560	12,000	34,311	-	55,000
Hg	-	0.1	-	-	0.4	0.2	0.1	-	<0.10	<0.10	<0.05	<0.05	<0.06	<0.06	0.1	0.5	-	11
Li	-	-	-	-	-	-	-	-	4	2	4	3	19	16	10	38	-	160
Mg	-	-	-	-	-	-	-	-	3184	2119	1411	1312	11,760	13,880	-	36,049	-	-
Mn	-	-	3249	1620	-	-	-	631	524	137	89	81	321	290	440	1212	-	0
Mo	-	-	-	-	-	-	-	-	<5.0	<5.0	<1.0	<1.0	<2.5	<2.2	-	5	-	390
Na	-	-	-	-	-	-	-	-	125	101	<88	51	114	178	-	519	-	-
Ni	54	40	158	37	40	40	25	36	9	4	8	5	23	21	20	55	15,000	840
Pb	171	137	104	102	256	135	105	102	12	9	6	3	9	8	21	39	-	400
Sb	-	-	-	-	-	-	-	-	-	-	<0.33	<0.30	<0.52	<0.40	-	12	-	31
Se	-	1.5	-	-	-	1.4	1.1	-	<0.5	<0.50	<0.40	<0.34	<0.50	<0.50	0.4	1.3	-	390
Sr	-	-	462	265	-	-	-	199	-	106	28	12	102	100	-	150	-	47,000
Ti	-	-	1080	2468	-	-	-	2599	95	127	150	117	100	120	MNL	208	-	47,000
Tl	-	-	-	-	-	-	-	-	<1.0	<1.0	<0.50	<0.50	<0.56	<0.50	-	3	-	2
V	-	-	-	-	-	-	-	-	21	15	10	8	23	21	-	60	-	390
Zn	-	587	1079	380	-	236	206	275	40	21	24	12	44	34	47	118	-	23,000

**Table 3.** Correlation coefficients between Detroit road dust and soil profiles and 8 literature profiles from the SPECIATE database [25]. *p*-value in parentheses. Red and yellow shading show statistical significance at *p* < 0.05 and *p* < 0.10 levels, respectively.

		Literature Profiles															
Detroit Profile	Type	Unpaved Road Dust		Industrial Soil		Sand & Gravel		Sandblast		Electric Arc Furnace		Auto Body Shredding		Fly Ash		Tire Dust	
Road dust—Source Area	Pearson	0.43	(0.11)	0.66	(0.01)	0.54	(0.04)	0.50	(0.06)	0.34	(0.21)	0.19	(0.49)	0.14	(0.63)	0.47	(0.08)
	Spearman	0.56	(0.03)	0.69	(0.00)	0.34	(0.22)	0.49	(0.06)	0.40	(0.14)	0.48	(0.07)	0.35	(0.21)	0.54	(0.04)
Road dust—Other Areas	Pearson	−0.02	(0.93)	0.20	(0.48)	−0.20	(0.47)	−0.17	(0.55)	0.02	(0.95)	0.14	(0.63)	0.17	(0.54)	0.08	(0.79)
	Spearman	0.55	(0.03)	0.70	(0.00)	0.15	(0.58)	−0.06	(0.83)	0.43	(0.11)	0.66	(0.01)	0.50	(0.06)	0.37	(0.18)
Background Soils	Pearson	0.01	(0.96)	0.13	(0.65)	−0.23	(0.41)	−0.17	(0.54)	0.07	(0.80)	0.03	(0.92)	0.08	(0.78)	0.10	(0.73)
	Spearman	0.36	(0.18)	0.33	(0.23)	−0.01	(0.97)	−0.16	(0.57)	0.33	(0.22)	0.34	(0.21)	0.31	(0.26)	0.20	(0.47)

**Table 4.** Enrichment ratios for road dust and soils in this study (“Detroit Samples”) and for literature profiles from the SPECIATE database. The heat map shows enrichment (red) and depletion (blue) from the crustal average.

	Detroit Samples			Literature Profiles							
Element	Road dust—Source Area	Road dust—Other Areas	Background Soils	Unpaved Road Dust	Industrial Soil	Sand & Gravel	Sandblast	Electric Arc Furnace	Auto Body Shredding	Fly Ash	Tire Dust
Al	0.2	0.7	1.0	0.7	0.4	1.2	0.8	0.2	0.1	0.8	0.0
Ca	2.9	2.3	1.1	1.8	3.6	0.5	0.2	0.3	6.7	5.3	0.0
Cr	6.6	1.3	1.0	1.9	5.7	1.9	52.4	4.0	6.1	3.4	0.3
Cu	4.8	1.0	0.8	1.7	3.0	1.1	7.6	5.5	2.4	8.5	7.4
Fe	5.1	0.7	0.7	0.9	0.6	0.7	1.4	0.5	4.7	1.1	0.1
K	0.2	0.6	0.7	0.8	0.4	0.8	0.5	3.8	1.4	0.3	0.0
Mn	3.4	1.6	0.6	1.2	2.6	1.4	1.5	NA	2.9	0.9	0.1
Ni	2.0	0.4	0.4	0.6	0.6	0.8	42.7	NA	2.1	1.3	0.7
P	2.4	9.5	5.1	0.8	2.2	0.5	0.5	1.8	3.3	3.6	1.8
Pb	1.3	1.3	1.3	3.9	2.5	0.5	6.8	17.5	8.1	5.9	2.2
S	1.5	2.4	1.1	1.6	5.2	1.1	5.5	8.2	17.1	14.8	6.5
Si	0.5	1.2	1.3	0.7	0.5	0.9	1.3	0.2	0.1	0.3	0.0
Sr	1.9	1.1	0.8	1.2	1.3	0.9	1.6	NA	4.7	3.7	0.3
Ti	0.2	0.5	0.5	0.7	0.6	0.5	0.9	0.1	0.4	1.8	0.1
Zn	6.0	2.1	1.5	4.1	7.1	1.3	18.4	26.6	5.2	4.0	30.1

## Data Availability

Data supporting the reported results can be obtained from the corresponding author (S.B.)
