# Supplementary material for: Fugitive Dust Associated with Scrap Metal Processing
Source: Environments. Author manuscript; Available in PMC 2026 Jul 8. (PMC13341166; doi:10.3390/environments10120223)
Supplement: Supplementary Material [file NIHMS2189907-supplement-Supplementary_Material.pdf]

Fugitive dust associated with scrap metal processing

Jeff Gearhart, Tian Xia, Md Kamrul Islam, Albert Shim, Sung-Hee Seo, Melissa Cooper Sargent, Natalie R. Sampson, Jacob Napieralski, Simone Sagovac, Stuart Batterman

Supplemental Information

Normalization of XRF measurements

Accurate XRF measurements require that samples completely fill the XRF sensing field (both aperture and depth). This condition may be unmet by wipe samples considering the small amount of dust collected. Significant and positive correlation between sample weight and elemental measurements (in ppm; Spearman rank correlation coefficients from 0.50 to 0.66 for Ca, Cr, Cu, Fe, K, Mn, Ni, Ti and Zn) demonstrate this concern, showing that concentrations were not independent of sample weight. This was not a concern for the soil samples, which completely filled the sample cassette.

Several approaches might be used to normalize the XRF data. First, dividing by sample weight would express concentration in unusual units (e.g., ppm/g or $\mu g/g^2$) and remains unlikely to fully compensate for loading effects. Moreover, sample weights were unavailable for 9 samples, and low sample weights (below ~20 mg) were uncertain given the variation in the tare weight. Second, dividing by the sampled area (giving $\mu g/g\text{-}cm^2$) might be useful if areas were similar, but this was not always the case. Third, XRF data might be normalized to a common element (e.g., Al, Ca, Fe or Si). However, levels of these elements were variable, especially in the wipe samples as many samples did not resemble native or natural soils (discussed later). We propose a fourth approach, normalizing by the reconstructed mass (RM), calculated as the sum of the common oxidized form of minerals based on XRF measurements. The RM often is used to provide a quality check and the mass estimate for speciated air samples (Chow et al., 2015). This has the advantage over the third approach of utilizing all XRF data and not depending on a single elemental measurement. A recommended RM equation is:

$$RM = \text{reconstructed mass} = 2.2\,Al + 1.63\,Ca + 2.42\,Fe + 2.49\,Si + 1.94\,Ti + \Sigma_i E_i \quad (1)$$

where Al, Ca, Fe, Si and Ti are XRF measurements for these elements (ppm), E_i are other XRF measurements (ppm), and the coefficients represent stoichiometric ratios for the main oxidized form. Across the wipe samples, RM averaged only 37,800 ppm (range: 1,200 to 159,900). Each XRF determination was divided by sample's RM, forming the reconstructed mass fraction for each element F_{Ei} (dimensionless, e.g., g/g):

$$F_{Ei} = E_i / RM \quad (2)$$

Finally, summing across elements gives the weight fraction of measured constituents, called Σ_F (dimensionless):

$$\Sigma_F = \Sigma_i F_{Ei} \quad (3)$$

RM and Σ_F were computed for each sample. Σ_F did not vary significantly by the type of wipe sample, e.g., Σ_F averaged 0.498 (N=17) for window samples, 0.478 (N=15) for painted surfaces, and 0.474 (N=13) for other wipe samples. The missing fraction (1 - Σ_F) likely is various forms of carbon (elemental and organic) and elements below the limit of detection. As expected, Σ_F was higher, 0.707, for the soil and road dust samples (N=20) that were likely to contain less carbon. While not reaching unity (i.e., accounting for 100% of the composition), Σ_F was considerably higher than sum of the XRF measurements. Σ_F had slight negative correlation with sample weight and no strong trend. Importantly, using F_{Ei} instead of the direct measurement E_i nearly halved the variation of measurements for each element with the exception of Cu, however, removing a single Cu data point gave a comparable reduction. (The outlier was sample R34, a wipe sample collected on a fire hydrant that had the highest levels of Cu, Fe and Zn among wipe samples.) Further, correlations and other statistics were considerably more consistent using F_{Ei}, e.g., pairs of metals such as Cu and Zn, Mn and Ni, Fe and Mn, and Fe and Ni were strongly correlated, as expected. A final advantage is that both wipe and soil/dust samples can use this normalization, thus allowing for direct comparison. For these reasons, normalization by reconstructed mass appears beneficial, although biases are possible if the mass fraction of an element used to identify a

source was both strongly correlated to the RM and constituted a large fraction of the mass (e.g., ≥30%). Spearman correlation coefficients between mass fractions and reconstructed mass were low with the exception of Ni (R=0.58). Because metals of most interest had small mass fractions (<0.001) except for Fe (described in the text), bias seems unlikely.

Ambient PM measurements by MPAL

The Michigan Pollution Assessment Laboratory (MPAL) is a mobile air quality monitoring platform that measures various particulate matter and gaseous air pollutants. The Horiba PX-375 (Horiba, Kyoto, Japan) installed onboard measures 1-minute PM_{10} using β-attenuation; and 30-minute PM_{10} metals and other elements, including Ti, V, Cr, Mn, Fe, Ni, Cu, Zn, As, Pb, Al, Si, S, K, and Ca, using X-ray fluorescence (XRF) spectrometry. In addition to Horiba data, this study examined the 1-min PM_{10} measured by another onboard instrument, the optical particle sizer (OPS, TSI 3330, TSI Inc., Shoreview, MN, USA), and the wind direction and speed measured by MPAL's weather station (Young 92000, R.M. Young Company, Traverse City, MI, USA). MPAL was parked on Kaier Street opposite the metal processor on two days for three 30-min sampling periods (Table S2). While parked, the onboard rear-facing wide-angle camera (Spy Tec Mobius action camera) took time-lapse photos for every 2 s, which captured the traffic on Dearborn Street and the gate to the metal processor facility. The photos were reviewed to estimate the number of trucks traveled on Dearborn Street and among them, the number of trucks entering the facility.

In the three 30-min periods, the wind direction was SW to WSW, and the wind speed was relatively low (0.9 – 2.5 m/s). On 8/16/2019 a total of 53 trucks traveled on Dearborn Street over one hour, while on 2/14/2020, only 17 trucks were seen in 30 minutes. The number of trucks entering the facility was similar, about 12 trucks/hr.

Elevated PM_{10} was observed on 8/16/2019 but not on 2/14/2020, possibly due to the fewer traffic and wet ground surface (after snow) on 2/14/2020. Figure S5 shows that, after 40-min forward adjustment, PM_{10} measured by the Horiba had similar trends as compared to the OPC data, while the OPC were more sensitive, and the peak values were 5 – 10 times larger.

Fe concentration varied 0.6 – 5.4 $\mu g/m^3$ over the three 30-min period (Table S2), and the measurements over the entire sampling days were tabulated in Table S3. If a 30-min forward adjustment is applied, Fe concentration was consistently at ~ 1 $\mu g/m^3$ on 8/16/2019 and 0.3 $\mu g/m^3$ on 2/14/2020. The detected Fe may be from the metal processor, but could also be from a large construction site, as a larger peak was always detected near the construction site right before the parking period. The second peak on 2/14/2020 (14:00 – 15:00) was detected near the Dearborn monitoring site, which agreed with the site data analysis in Table S1.

In summary, the ambient measurements by MPAL detected elevated PM_{10} and Fe in southwest Detroit. Potential sources of Fe in PM_{10} include a large construction site, the metal processing facility, and the steel mill and other industries near the Dearborn monitoring site. Longer and systematic sampling by MPAL at these locations is needed to have a representative source estimation.

Figure S1. 1949 arial view of processor. Note homes line south side of Dearborn street and only the western portion of the property appears to be industrial. From https://digital.library.wayne.edu/dte_aerial/part2/wayne/1949/ha-18-99.pdf.

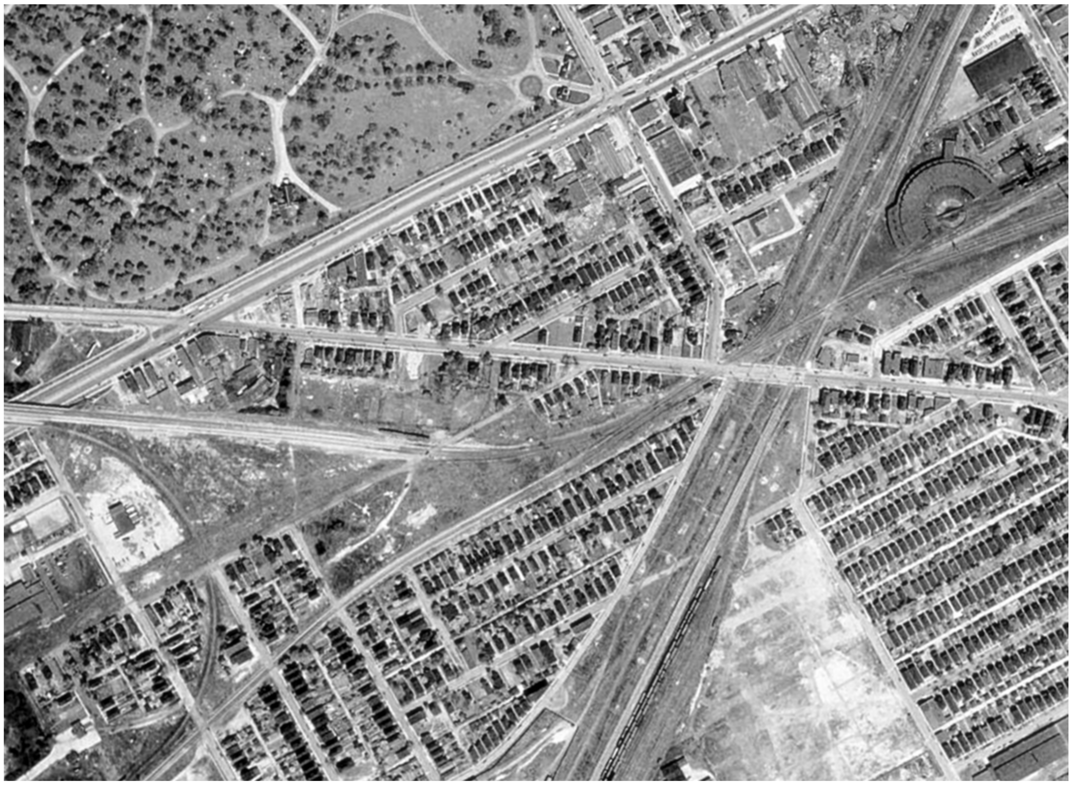

Figure S2. Photos showing arial view of facility. Top: Looking west.   Bottom: Looking East.

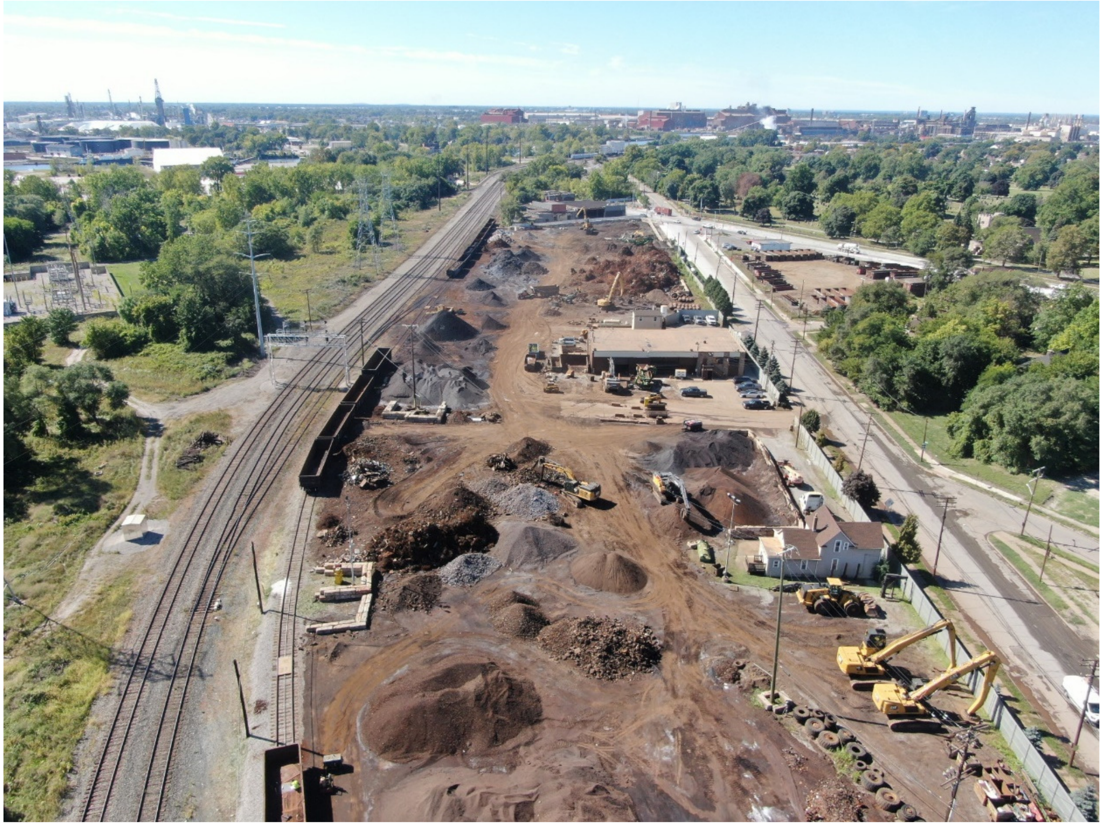

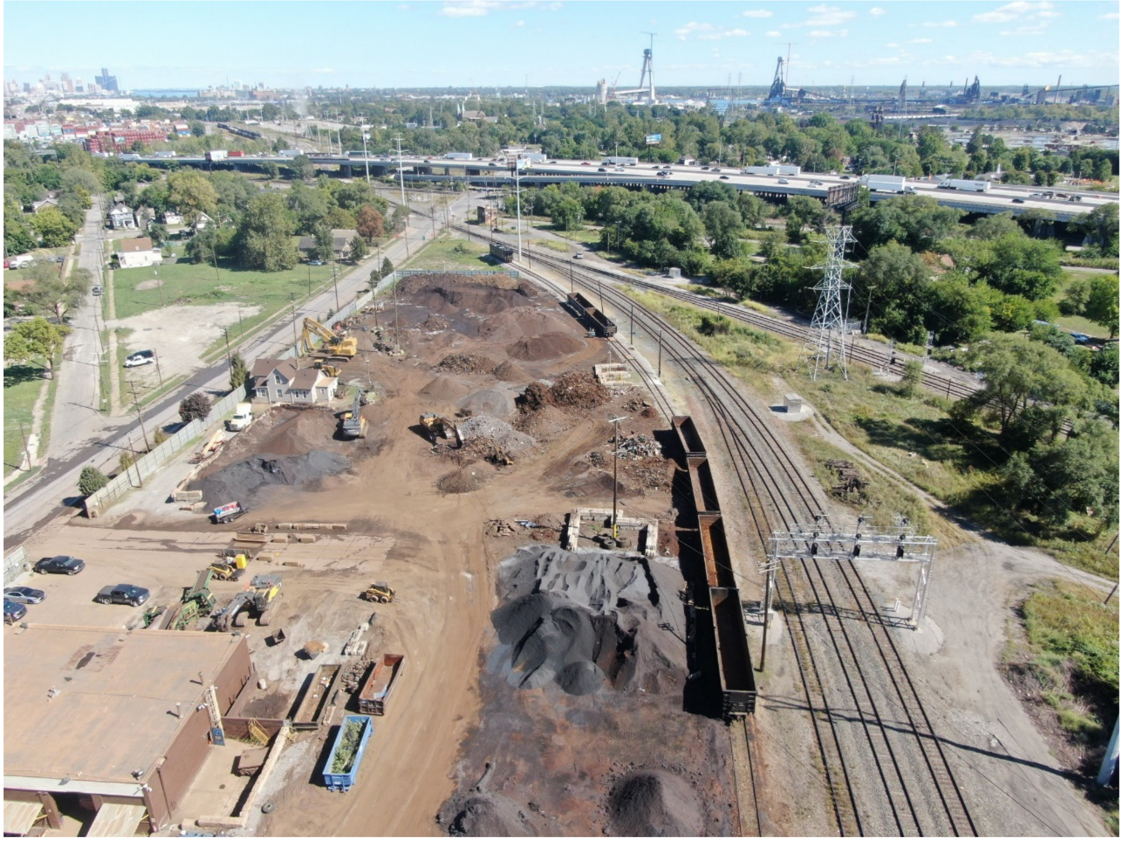

Figure S3. Photos showing examples of wipe sampling locations. 1st row: Porch, tile and window sampling along Dearborn Street. 2nd row: plastic table, window and glass table in Kaier Street area. 3rd row: window, plexiglass sheet and back yard window on Graham Street area. 4th row: window, car windshield and glass plate in Graham Street area.

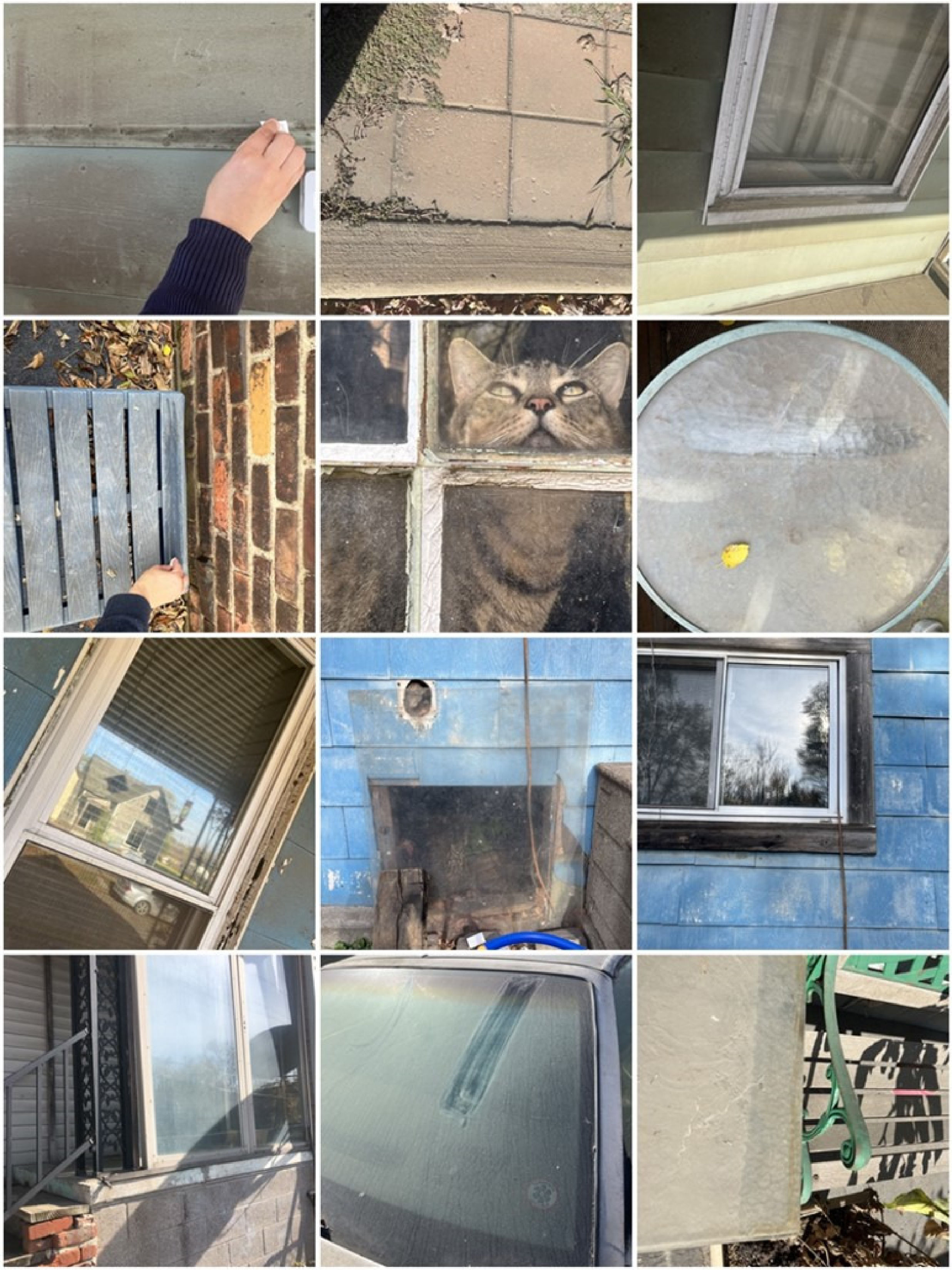

Figure S4. Comparison of ambient concentrations of available metals in TSP at the Dearborn monitoring site (annual average for 2021) to soil/dust mass fractions for road dust in the source area, road dust in other areas, and background soils. Line shows average ratio between TSP concentration and soil/dust mass fraction for Cu and Zn, which is 64196, 232865, and 306492 for the three figures (from left to right). Equal ratios would lay on lines parallel to that displayed.

The figures suggest that relative to ambient TSP, soils and dusts are enriched in Cu and Zn (and Pb and Mn for the source area) compared to the other elements (e.g., Fe, Mn, Cr). This analysis has limitations: the TSP metals were collected at a different site; only a subset of metals are included in the TSP measurements; only 2021 data are used for the TSP. Similar analyses for PM_{10} and $PM_{2.5}$ show considerably more scatter, indicating greater variation between metal compositions in dusts/soils and ambient air for the smaller size fraction. This is consistent with soils and dusts reflecting primarily dust fall of larger particles.

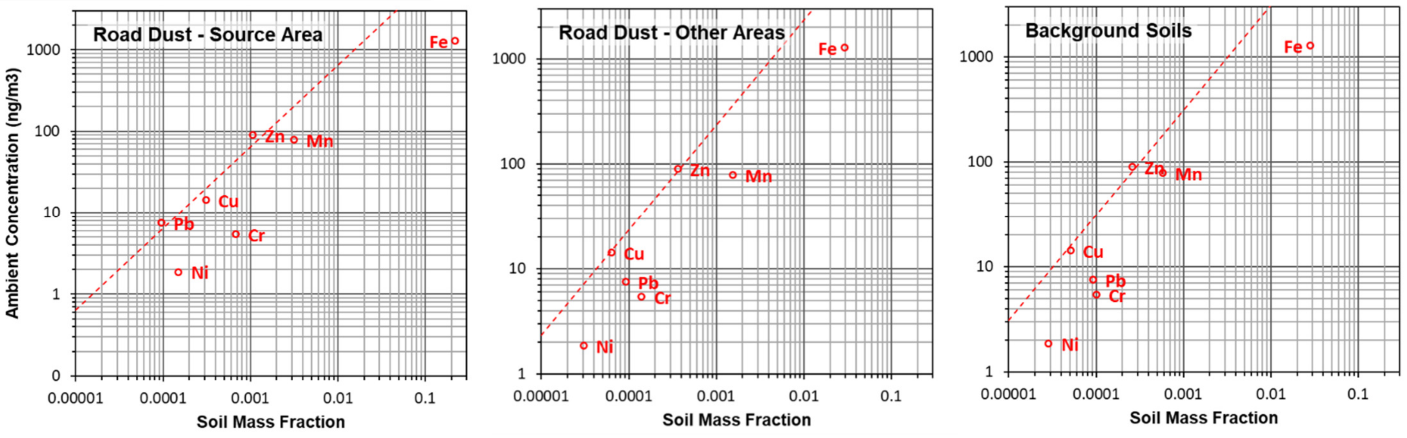

Table S1. Annual average concentrations of selected elements (ng/m^3) and PM (µg/m^3) in ambient air in three size fractions at local monitoring sites for 2021. Sites are Trinity St. Marks, New Mount Herman, Military Park, Southwest High School, Dearborn, Detroit Precinct 4, and River Rouge. From US EPA Air Data Air Quality Monitors, https://epa.maps.arcgis.com/apps/webappviewer.

	TSP							PM10							PM2.5						
Element	TRIN	NMH	MPARK	SWHS	DB	DP4	RIVERR	TRIN	NMH	MPARK	SWHS	DB	DP4	RIVERR	TRIN	NMH	MPARK	SWHS	DB	DP4	RIVERR
As	2.0	1.2	1.7	1.5	1.3	1.2	1.2	-	-	-	-	1.127	-	-	-	-	-	0.0	0.0	-	-
Cd	0.2	0.1	0.2	0.2	0.3	0.1	0.4	-	-	-	-	0.196	-	-	-	-	-	0.6	0.8	-	-
Al	-	-	-	-	-	-	-	-	-	-	-	-	-	-	-	-	-	38.2	35.4	-	-
Ca		-	-	-	-	-	-	-	-	-	-	-	-	-	-	-	-	118.1	100.6	-	-
Ce		-	-	-	-	-	-	-	-	-	-	-	-	-	-	-	-	0.4	0.1	-	-
Cr		-	-	-	5.4	-	-	-	-	-	-	3.3	-	-	-	-	-	1.4	2.3	-	-
Cu		-	-	-	14.1	-	-	-	-	-	-	14.0	-	-	-	-	-	14.0	19.4	-	-
Fe		-	-	-	1269.9	-	-	-	-	-	-	702.1	-	-	-	-	-	111.0	249.4	-	-
K		-	-	-	-	-	-	-	-	-	-	-	-	-	-	-	-	66.1	86.3	-	-
La		-	-	-	-	-	-	-	-	-	-	-	-	-	-	-	-	-	-	-	-
Mn	67.2	20.8	50.1	51.6	77.6	46.6	32.0	-	-	-	-	37.7	-	-	-	-	-	3.6	5.4	-	-
Ni	3.1	1.3	1.6	1.8	1.9	1.8	1.2	-	-	-	-	1.4	-	-	-	-	-	0.5	1.0	-	-
P		-	-	-	-	-	-	-	-	-	-	-	-	-	-	-	-	0.2	0.1	-	-
Pb	7.5	3.4	10.6	7.2	7.5	6.9	4.8	-	-	-	-	7.2	-	-	-	-	-	2.8	3.3	-	-
Rb		-	-	-	-	-	-	-	-	-	-	-	-	-	-	-	-	-	-	-	-
S		-	-	-	-	-	-	-	-	-	-	-	-	-	-	-	-	422.5	449.7	-	-
Si		-	-	-	-	-	-	-	-	-	-	-	-	-	-	-	-	97.7	79.8	-	-
Sr		-	-	-	-	-	-	-	-	-	-	-	-	-	-	-	-	1.0	1.2	-	-
Ti		-	-	-	-	-	-	-	-	-	-	-	-	-	-	-	-	3.5	3.2	-	-
Zn		-	-	-	88.9	-	-	-	-	-	-	69.6	-	-	-	-	-	21.5	34.7	-	-
PM		-	-	-	-	-	-	-	-	-	23.6	23.2	-	-	12.0	9.9	11.2	10.9	9.8	11.2	-

Table S2. PM_{10}, selected element, and other measurements by MPAL during the three 30-min periods. The number of trucks were estimated according to the time-lapse photos taken every 2 s by the onboard rear-facing wide-angle camera. Wind direction and speed were acquired by the onboard weather station.

Date	8/16/2019	8/16/2019	2/14/2020
Start	11:00	11:30	12:30
End	11:30	12:00	13:00
No. of trucks on Dearborn St.	29	34	17
No. of trucks entering the facility	6	7	5
PM_{10}_Horiba (□g/m^3)	47.3	81.6	30.2
PM_{10}_OPC (µg/m^3)	163.3	103.2	34.7
Fe (µg/m^3)	5.4	1.0	0.6
Si (µg/m^3)	1.3	0.8	0.5
S (µg/m^3)	0.6	0.6	-0.1
Ca (µg/m^3)	2.4	0.7	0.6
Wind Dir (°)	245.4	233.0	267.2
Wind Speed (m/s)	1.9	0.9	2.5

Table S3. PM_{10}, Fe and Ca concentrations measured by Horiba PX-375 over the entire sampling visits to Detroit on 8/16/2019 and 2/14/2020. The three 30-min periods when MPAL was parked near the metal processor were marked yellow.

Date&Time	PM_{10}(μg/m^3)	Fe(ng/m^3)	Ca(ng/m^3)
8/16/2019 9:00	13.3	559.1	486.5
8/16/2019 9:30	21.8	616.8	852.0
8/16/2019 10:00	20.0	1198.3	2152.3
8/16/2019 10:30	24.9	12057.4	5255.5
8/16/2019 11:00	47.3	5431.3	2358.1
8/16/2019 11:30	81.6	1005.8	740.6
8/16/2019 12:00	76.8	966.8	945.2
8/16/2019 12:30	44.2	1081.6	1723.2
8/16/2019 13:00	32.7	778.1	1273.3
8/16/2019 13:30	40.5	1529.4	1627.4
8/16/2019 14:00	39.8	801.8	671.3
2/14/2020 9:30	14.9	630.2	1203.3
2/14/2020 10:00	34.9	261.0	346.9
2/14/2020 10:30	48.6	319.5	824.0
2/14/2020 11:00	37.8	635.5	1772.8
2/14/2020 11:30	38.2	485.6	846.7
2/14/2020 12:00	41.3	1157.4	887.3
2/14/2020 12:30	30.2	555.2	590.6
2/14/2020 13:00	19.9	269.2	292.7
2/14/2020 13:30	15.7	481.2	575.1
2/14/2020 14:00	16.4	1873.9	5171.7
2/14/2020 14:30	28.0	1021.3	3231.5
2/14/2020 15:00	77.5	953.3	2270.3
2/14/2020 15:30	63.0	763.7	1603.9
2/14/2020 16:00	55.1	111.3	226.3
2/14/2020 16:30	37.0	46.2	-22.0
2/14/2020 17:00	7.7	26.4	-135.6

Figure S5. 1-minute PM_{10} concentration trends measured by Horiba and OPC over the entire sampling visits to Detroit on 8/16/2019 and 2/14/2020. The three 30-min periods when MPAL was parked near the metal processor were marked between the two dashed vertical lines. Horiba data were adjusted forward by 40 minutes to match the trends with OPC.

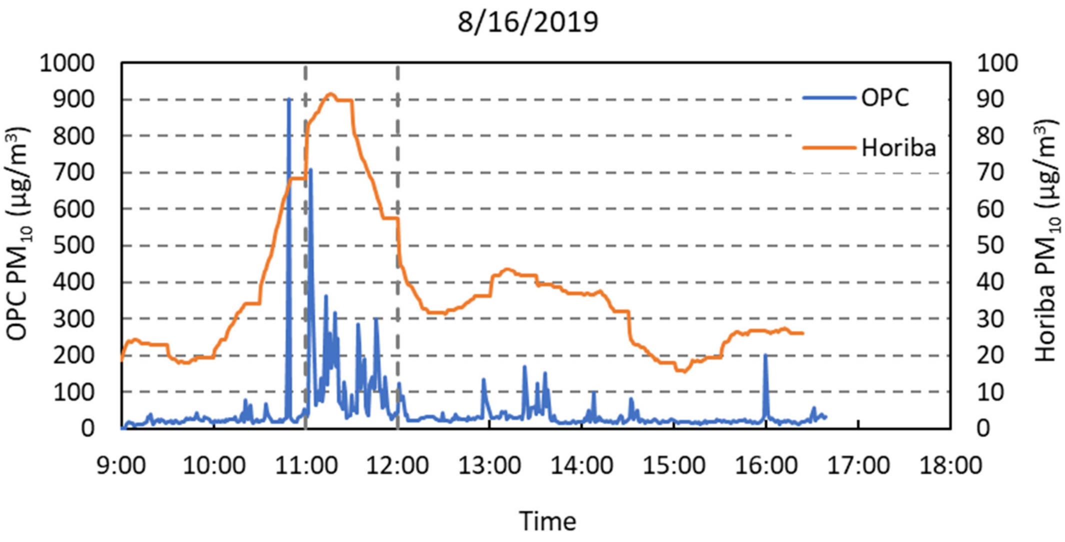

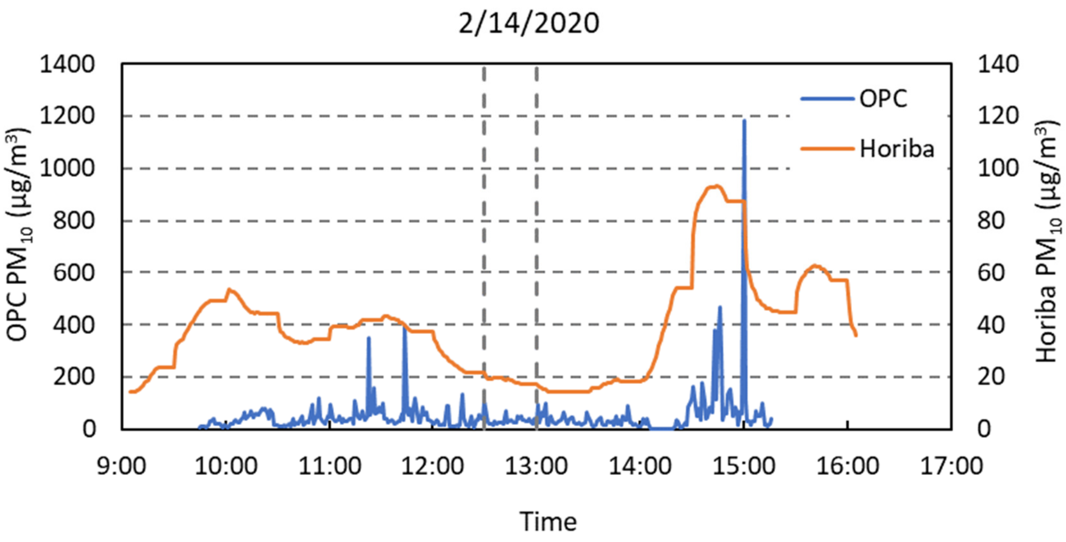

Table S4. List of metal processing firms identified in Detroit. Continues on several pages.

Name of Business or shop	Latitude	Longitude	Category	Reduced Category	Address	City/Area	State	ZIP
Arco Alloys Corp.	42.38	-83.05	Alloy	Alloy products & casting	1891 Trombly St	Detroit	Michigan	48211
Crown Alloys Company	42.51	-83.12	Alloy	Alloy products & casting	30105 Stephenson Hwy	Madison Heights	Michigan	48071
Metro Corporation	42.42	-83.06	Alloy	Alloy products & casting	17385 Ryan Rd	Hamtramck	Michigan	48212
SAS Global Corporation	42.46	-83.01	Alloy	Alloy products & casting	21601 Mullin Ave	Warren	Michigan	48089
Eutectic Engineering Co	42.42	-83.04	Aluminum Castings	Alloy products & casting	6350 E Davison St	Hamtramck	Michigan	48212
Metro Alloys	42.42	-83.06	Aluminum Castings	Alloy products & casting	17385 Ryan Rd		Michigan	48212
Super Steel Treating Co	42.47	-83.04	Annealing	Annealing, plating & coatings	6227 Rinke Ave	Warren	Michigan	48091
Weldaloy Specialty Forgings	42.47	-83.01	Annealing	Annealing, plating & coatings	24011 Hoover Rd	Warren	Michigan	48089
Western Engineered Products	42.82	-83.25	Annealing	Annealing, plating & coatings	465 S Glaspie St suite g	Oxford	Michigan	48371
2 New Detailing- Paint Correction & C	42.47	-82.95	Coatings	Annealing, plating & coatings	23450 Gratiot Ave	Eastpointe	Michigan	48021
AJAX Metal Processing, Inc	42.37	-83.02	Coatings	Annealing, plating & coatings	4651 Bellevue St	Detroit	Michigan	48207
A-Line Products Corporation	42.36	-83.02	Coatings	Annealing, plating & coatings	2955 Bellevue St	Detroit	Michigan	48207
All Cote Coatings Company	42.47	-83.04	Coatings	Annealing, plating & coatings	23896 Sherwood Ave	Center Line	Michigan	48015
Alpine Jig Grinding Company	42.34	-83.29	Coatings	Annealing, plating & coatings	25325 W Warren St	Dearborn Heights	Michigan	48127
Astral Products	42.57	-83.45	Coatings	Annealing, plating & coatings	3182 Martin Pkwy	Commerce Charter Twp	Michigan	48390
Commercial Painting Services	42.47	-83.24	Coatings	Annealing, plating & coatings	24700 Northwestern Hwy #50	Southfield	Michigan	48075
Creative Coatings Inc	42.47	-83.04	Coatings	Annealing, plating & coatings	24650 Mound Rd	Warren	Michigan	48091
Custom Concepts Powder Coating &	42.44	-83.04	Coatings	Annealing, plating & coatings	19330 Mt Elliott St	Detroit	Michigan	48234
Derick Jig Grinding Inc	42.34	-83.29	Coatings	Annealing, plating & coatings	25325 W Warren St	Dearborn Heights	Michigan	48127
Fabcoat Performance Coatings	42.22	-83.15	Coatings	Annealing, plating & coatings	210 Clark St #3	Wyandotte	Michigan	48192
Fitzgerald Finishing LLC	42.42	-83.04	Coatings	Annealing, plating & coatings	17450 Filer St	Detroit	Michigan	48212
Fletcher Paint (Fletcher Precision Ma	42.46	-83.03	Coatings	Annealing, plating & coatings	6795 E 9 Mile Rd	Warren	Michigan	48091
Fred's Bump & Paint Shop	42.39	-83.05	Coatings	Annealing, plating & coatings	2951 Council St	Hamtramck	Michigan	48212
G & T Commercial Coating	42.37	-83.29	Coatings	Annealing, plating & coatings	25650 Plymouth Rd	Redford Charter Twp	Michigan	48239
General Acid Proofing Inc	42.35	-83.01	Coatings	Annealing, plating & coatings	1051 Bellevue St	Detroit	Michigan	48207
International Hardcoat Inc.	42.38	-83.25	Coatings	Annealing, plating & coatings	12400 Burt Rd	Detroit	Michigan	48228
Maaco Auto Body Shop & Painting	42.37	-83.22	Coatings	Annealing, plating & coatings	18250 Plymouth Rd	Detroit	Michigan	48228
Magni Industries, Inc.	42.32	-83.11	Coatings	Annealing, plating & coatings	2771 Hammond St	Detroit	Michigan	48209
Metro Powder Coating	42.37	-83.22	Coatings	Annealing, plating & coatings	18434 Fitzpatrick St	Detroit	Michigan	48228
MOSTEK PAINT AND GLASS CO	42.40	-83.06	Coatings	Annealing, plating & coatings	11515 Joseph Campau Ave	Hamtramck	Michigan	48212
Painters Supply & Equipment Co.	42.46	-83.03	Coatings	Annealing, plating & coatings	22134 Van Dyke Ave	Warren	Michigan	48089
PPG Paint Store & Paint Sprayer Re	42.46	-83.28	Coatings	Annealing, plating & coatings	23361 Telegraph Rd	Southfield	Michigan	48034
Supreme Welding-Powder Coating	42.55	-82.88	Coatings	Annealing, plating & coatings	34727 Nova Dr	Clinton Twp	Michigan	48035
United Paint & Chemical Corporation	42.47	-83.28	Coatings	Annealing, plating & coatings	24671 Telegraph Rd	Southfield	Michigan	48033
Westside Powder Coat LLC	42.19	-83.39	Coatings	Annealing, plating & coatings	35777 Genron Ct	Romulus	Michigan	48174
Artistic Iron Works	42.29	-83.15	Iron and Iron Work	Ferrous processing & fabricat	420 S Oakwood	Detroit	Michigan	48217
Fort Iron And Metal	42.29	-83.13	Iron and Iron Work	Ferrous processing & fabricat	9607 Dearborn St	Detroit	Michigan	48209
Judd Industrial Contracting Inc	42.43	-83.06	Iron and Iron Work	Ferrous processing & fabricat	17900 Ryan Rd	Detroit	Michigan	48212
McHugh Iron And Steel Corp	42.33	-83.08	Iron and Iron Work	Ferrous processing & fabricat	2400 20th St	Detroit	Michigan	48216
Nelson Iron Works Inc	42.39	-83.04	Iron and Iron Work	Ferrous processing & fabricat	6350 Benham St	Detroit	Michigan	48211
Acme Wire & Iron Works	42.37	-83.03	Iron and Iron Work	Ferrous processing & fabricat	3527 E Canfield St	Detroit	Michigan	48207
Artistic Disenos, Inc	42.31	-83.12	Iron and Iron Work	Ferrous processing & fabricat	6789 Goldsmith St	Detroit	Michigan	48209
Bar Processing Warren	42.46	-82.99	Iron and Iron Work	Ferrous processing & fabricat	22534 Groesbeck Hwy	Warren	Michigan	48089
Detroit Nipple Works	42.37	-83.07	Iron and Iron Work	Ferrous processing & fabricat	6530 Beaubien Blvd	Detroit	Michigan	48202
EJ - Detroit Branch	42.45	-83.17	Iron and Iron Work	Ferrous processing & fabricat	13001 Northend Ave	Oak Park	Michigan	48237
Ferrous Processing & Trading	42.41	-83.04	Iron and Iron Work	Ferrous processing & fabricat	13200 Mt Elliott St	Hamtramck	Michigan	48212
Ferrous Processing & Trading Co Ink	42.29	-83.33	Iron and Iron Work	Ferrous processing & fabricat	29131 Michigan Ave	Inkster	Michigan	48141
Ferrous Processing & Trading Co Wy	42.31	-83.16	Iron and Iron Work	Ferrous processing & fabricat	3651 Wyoming Ave	Dearborn	Michigan	48120
Ferrous Processing & Trading Co.	42.34	-83.04	Scrap	Ferrous processing & fabricat	1333 Brewery Park Blvd	Detroit	Michigan	48207
Jefferson Iron Works Inc	42.47	-83.13	Iron and Iron Work	Ferrous processing & fabricat	2441 Wolcott St	Ferndale	Michigan	48220
Jim's Iron & Aluminum	42.35	-83.20	Iron and Iron Work	Ferrous processing & fabricat	7535 Greenfield Rd	Detroit	Michigan	48228
Metal City Fab	42.45	-82.99	Iron and Iron Work	Ferrous processing & fabricat	21200 Schoenherr Rd	Warren	Michigan	48089
Nelson Iron Works Inc	42.39	-83.04	Iron and Iron Work	Ferrous processing & fabricat	6350 Benham St	Detroit	Michigan	48211
Aluminum Supply Co Inc	42.39	-83.17	Metal Fabricators	Metal fabricator	14359 Meyers Rd	Detroit	Michigan	48227
Aluminum & Architectural Metals	42.36	-83.13	Metal Fabricators	Metal fabricator	8711 Epworth St	Detroit	Michigan	48204
Anderzaks Machine & Fab LLC	42.38	-83.28	Metal Fabricators	Metal fabricator	24607 Capitol Redford	Redford	Michigan	48239
Boyer Steel	42.44	-83.11	Metal Fabricators	Metal fabricator	19640 Charleston St	Highland Park	Michigan	48203
Detroit Fab	42.43	-83.04	Metal Fabricators	Metal fabricator	18480 Mt Elliott St	Detroit	Michigan	48234
Fabrications Unlimited Inc	42.37	-83.02	Metal Fabricators	Metal fabricator	4651 Beaufait St	Detroit	Michigan	48207
Franklin Fastener	42.37	-83.30	Metal Fabricators	Metal fabricator	12071 Beech Daly	Redford Charter Twp	Michigan	48239
GANAS MFG	42.36	-83.02	Metal Fabricators	Metal fabricator	3250 Bellevue St	Detroit	Michigan	48207
H M White Inc	42.38	-83.25	Metal Fabricators	Metal fabricator	12855 Burt Rd	Detroit	Michigan	48223
J & S Architectural Sheet Metal Inc	42.44	-83.08	Metal Fabricators	Metal fabricator	20137 Dequindre St	Detroit	Michigan	48234
J Max Robertson Co	42.40	-83.17	Metal Fabricators	Metal fabricator	12811 Hillview Street	Detroit	Michigan	48227
Lawford Fabricating Co	42.45	-83.16	Metal Fabricators	Metal fabricator	21650 Wyoming Ct	Oak Park	Michigan	48237
Metal Fab Inc.	42.34	-83.19	Metal Fabricators	Metal fabricator	6900 Chase Rd	Dearborn	Michigan	48126

Milton Manufacturing Inc	42.43	-83.10	Metal Fabricators	Metal fabricator	301 E Grixdale	Detroit	Michigan	48203
PK Fabricating	42.46	-83.12	Metal Fabricators	Metal fabricator	1975 Hilton Rd	Ferndale	Michigan	48220
RAMCO - Reliable Architectural Meta	42.40	-83.02	Metal Fabricators	Metal fabricator	9751 Erwin Ave	Detroit	Michigan	48213
Smith Shop	42.41	-83.09	Metal Fabricators	Metal fabricator	180 Victor St	Highland Park	Michigan	48203
Songer Steel Services	42.29	-83.11	Metal Fabricators	Metal fabricator	1400 Zug Island Rd	Detroit	Michigan	48209
Arted Chrome Plating Inc	42.37	-83.07	Metal Finishers	Annealing, plating & coatings	38 Piquette St	Detroit	Michigan	48202
Automotive Tumbling Company	42.36	-83.02	Metal Finishers	Annealing, plating & coatings	3125 Meldrum St	Detroit	Michigan	48207
Dci Aerotech Division Of Detroit Chro	42.40	-83.15	Metal Finishers	Annealing, plating & coatings	7515 Lyndon St	Detroit	Michigan	48238
Fitzgerald Finishing Co	42.42	-83.04	Metal Finishers	Annealing, plating & coatings	17450 Filer St	Hamtramck	Michigan	48212
Liberty Burnishing Co	42.43	-83.03	Metal Finishers	Annealing, plating & coatings	18401 Sherwood St	Detroit	Michigan	48234
Bellevue Processing Corp	42.37	-83.02	Metal Heat Treating	Heat treating	5143 Bellevue St	Detroit	Michigan	48211
Can Am	42.42	-83.07	Metal Heat Treating	Heat treating	2731 Jerome St	Detroit	Michigan	48212
Cooper Heat Treating LLC	42.45	-83.03	Metal Heat Treating	Heat treating	20251 Sherwood St	Detroit	Michigan	48234
Detroit Flame Hardening Co	42.42	-83.04	Metal Heat Treating	Heat treating	17644 Mount Elliott St	Hamtramck	Michigan	48212
Federal Screw Works	42.33	-83.05	Metal Heat Treating	Heat treating	535 Griswold St	Detroit	Michigan	48226
Fire Rite Inc	42.39	-83.18	Metal Heat Treating	Heat treating	13801 Lyndon St	Detroit	Michigan	48227
Hy Vac Technologies Incorporated	42.38	-83.20	Metal Heat Treating	Heat treating	15701 Glendale St	Detroit	Michigan	48227
Kamax Gb Dupont Lp Hamtramck	42.39	-83.05	Metal Heat Treating	Heat treating	3120 Denton St	Hamtramck	Michigan	48211
Superior Heat Treat Inc	42.40	-83.01	Metal Heat Treating	Heat treating	9770 Grinnell St	Detroit	Michigan	48213
Aalberts surface technologies - HIP |	42.37	-83.41	Metal Heat Treating	Heat treating	12238 Newburgh Rd	Livonia	Michigan	48150
Almar Industries Inc	42.45	-83.08	Metal Heat Treating	Heat treating	21005 Dequindre Rd #2610	Hazel Park	Michigan	48030
American Metal Processing Co	42.46	-83.00	Metal Heat Treating	Heat treating	22720 Nagel St	Warren	Michigan	48089
Apollo Heat Treating	42.46	-83.17	Metal Heat Treating	Heat treating	10400 Capital St	Oak Park	Michigan	48237
Bodycote Thermal Processing	42.38	-83.36	Metal Heat Treating	Heat treating	31888 Glendale St	Livonia	Michigan	48150
Cooper Heat Treating LLC	42.45	-83.04	Metal Heat Treating	Heat treating	20251 Sherwood St	Detroit	Michigan	48234
Detroit Steel Treating Co	42.68	-83.29	Metal Heat Treating	Heat treating	1631 E Highwood	Pontiac	Michigan	48340
East-Lind Heat Treat Inc	42.53	-83.09	Metal Heat Treating	Heat treating	32045 Dequindre Rd	Madison Heights	Michigan	48071
Engineered Heat Treat, Inc.	42.52	-83.12	Metal Heat Treating	Heat treating	31271 Stephenson Hwy	Madison Heights	Michigan	48071
Fire-Rite Inc	42.39	-83.18	Metal Heat Treating	Heat treating	13801 Lyndon St	Detroit	Michigan	48227
Heat Treating Services	42.62	-83.28	Metal Heat Treating	Heat treating	217 Central Ave	Pontiac	Michigan	48341
Hy-Vac Technologies Inc	42.38	-83.20	Metal Heat Treating	Heat treating	15701 Glendale St	Detroit	Michigan	48227
Induction Services Inc	42.48	-83.04	Metal Heat Treating	Heat treating	24800 Mound Rd	Warren	Michigan	48091
Induction. Processing	42.48	-83.00	Metal Heat Treating	Heat treating	24872 Gibson Dr	Warren	Michigan	48089
Nitro-Vac Heat Treat Inc.	42.46	-83.08	Metal Heat Treating	Heat treating	23080 Dequindre Rd	Warren	Michigan	48091
Nor-Cote Inc	42.47	-83.01	Metal Heat Treating	Heat treating	11425 Timken Ave	Warren	Michigan	48089
Precision Steel Treating Co	42.42	-83.04	Metal Heat Treating	Heat treating	17635 Mt Elliott St	Detroit	Michigan	48212
Specialty Steel Treating Inc	42.55	-82.94	Metal Heat Treating	Heat treating	34501 Commerce Rd	Fraser	Michigan	48026
Superior Heat Treat LLC	42.56	-82.92	Metal Heat Treating	Heat treating	36125 Groesbeck Hwy	Clinton Twp	Michigan	48035
Peerless Metal Powders Incorporated	42.32	-83.11	Metal Powder Fabricating	Metal fabricator	124 S Military St	Detroit	Michigan	48209
Aluminum Supply Co Inc	42.39	-83.17	Metal Products	Metal fabricator	14359 Meyers Rd	Detroit	Michigan	48227
Detroit Tube Products	42.31	-83.09	Metal Products	Metal fabricator	300 S Junction St	Detroit	Michigan	48209
H & H Metals Co	42.29	-83.33	Metal Products	Metal fabricator	29131 Michigan Ave	Inkster	Michigan	48141
Shr Development Inc	42.38	-83.24	Metal Products	Metal fabricator	20539 Glendale St	Detroit	Michigan	48223
Sikora Metals	42.41	-83.04	Metal Products	Metal fabricator	12850 Mount Elliott Street	Detroit	Michigan	48212
Steel Services	42.32	-83.12	Metal Products	Metal fabricator	3401 Martin St	Detroit	Michigan	48210
Vulcanmasters Welding	42.29	-83.15	Metal Products	Metal fabricator	374 S Fordson St	Detroit	Michigan	48217
Winston Brothers Iron & Metal Comp	42.42	-83.07	Metal Products	Metal fabricator	17384 Conant St	Detroit	Michigan	48212
Alro Steel, Detroit	42.43	-83.04	Metal Products	Metal fabricator	18695 Sherwood St	Detroit	Michigan	48234
Detroit Architectural Metal	42.38	-83.29	Metal Products	Metal fabricator	25501 Glendale	Redford Charter Twp	Michigan	48239
Inter-City Steel Company	42.42	-83.07	Metal Products	Metal fabricator	17901 Conant	Detroit	Michigan	48212
Metal Design & Manufacturing	42.43	-83.06	Metal Products	Metal fabricator	17891 Ryan Rd	Detroit	Michigan	48212
Quality Bending & Threading	42.35	-83.09	Metal Products	Metal fabricator	5100 Stanton St	Detroit	Michigan	48208
Meccom Corporation	42.34	-83.14	Metal Rolling and Forming	Metal fabricator	5945 Martin St	Detroit	Michigan	48210
Meccom Inc	42.34	-83.14	Metal Rolling and Forming	Metal fabricator	5945 Martin St	Detroit	Michigan	48210
American Modern Roofing	42.40	-83.18	Metal Roofing and Siding	Metal fabricator	14916 Schaefer Hwy	Detroit	Michigan	48227
Cass Sheet Metal	42.39	-82.98	Metal Roofing and Siding	Metal fabricator	5641 Conner St	Detroit	Michigan	48213
Coil Slitting	42.42	-83.06	Metal Slit and Shear	Metal fabricator	17155 Conant St	Hamtramck	Michigan	48212
Metro Shearing	42.36	-83.02	Metal Slit and Shear	Metal fabricator	3250 Bellevue St	Detroit	Michigan	48207
Advance Engineering Co	42.37	-83.28	Metal Stamping	Metal stamping	12025 Dixie	Redford	Michigan	48239
L & W Engineering	42.40	-83.08	Metal Stamping	Metal stamping	1200 Woodland St	Detroit	Michigan	48211
Martinrea Fabco Hot Stampings	42.38	-83.23	Metal Stamping	Metal stamping	19200 Glendale St	Detroit	Michigan	48223
Metro Stamping & Manufacturing Co	42.38	-83.31	Metal Stamping	Metal stamping	26955 Fullerton	Redford	Michigan	48239
Motor City Industries Inc	42.29	-83.12	Metal Stamping	Metal stamping	8955 Thaddeus St	Detroit	Michigan	48209
New Center Stamping Inc	42.37	-83.06	Metal Stamping	Metal stamping	1140 E Milwaukee St	Detroit	Michigan	48211
New Center Stamping Inc Fax	42.37	-83.06	Metal Stamping	Metal stamping	950 E Milwaukee St	Detroit	Michigan	48211
Wayne Stamping International	42.43	-83.10	Metal Stamping	Metal stamping	301 E Grixdale	Highland Park	Michigan	48203
Wedu Manufacturing Corp	42.37	-83.28	Metal Stamping	Metal stamping	12042 Woodbine	Hamtramck	Michigan	48239
Sherwood Prototype Inc.	42.41	-83.09	Metal Stamping	Metal stamping	124 Victor St #3130	Highland Park	Michigan	48203
Ferrous Processing & Trading Co Po	42.69	-83.30	Non-Ferrous	Scrap metals	500 Collier Rd	Auburn Hills	Michigan	48326

Kwik Paint Products	42.37	-83.06	Retail - Coatings	X	6040 Russell St	Detroit	Michigan	48211
PT Automotive Paints & Parts	42.33	-83.11	Retail - Coatings	X	4828 Michigan Ave	Detroit	Michigan	48210
Hammer Time True Value Hardware	42.42	-83.14	Retail - HW	X	17400 Livernois	Detroit	Michigan	48221
Baker's Gas & Welding Supplies	42.26	-83.18	Retail - Welding	X	1300 Howard St	Lincoln Park	Michigan	48146
Larry's Welding Supply	42.46	-82.98	Retail - Welding	X	13845 E 9 Mile Rd	Warren	Michigan	48089
Linde Welding Gas & Equipment Cen	42.33	-83.16	Retail - Welding	X	5825 Wyoming Ave	Dearborn	Michigan	48126
New Century Welding	42.46	-83.08	Retail - Welding	X	22621 Dequindre Rd	Hazel Park	Michigan	48030
Nortronic Co	42.45	-83.03	Retail - Welding	X	20210 Sherwood St	Detroit	Michigan	48234
Robinson Welding Supply	42.37	-83.36	Retail - Welding	X	31700 Plymouth Rd	Livonia	Michigan	48150
Roy Smith Company	42.45	-83.28	Retail - Welding	X	21348 Telegraph Rd	Southfield	Michigan	48033
Weld-Aid	42.42	-83.08	Retail - Welding	X	14650 Dequindre St	Detroit	Michigan	48212
Welders Welding Supply Co	42.27	-83.24	Retail - Welding	X	20643 Van Born Rd	Taylor	Michigan	48180
Novi Tool & Machine Inc	42.38	-83.28	Retail Machine	X	12202 Woodbine		Michigan	48239
Sherwin-Williams Automotive Finishe	42.40	-83.28	Retail Paints	X	15324 Telegraph Rd	Detroit	Michigan	48239
Sherwin-Williams Commercial Paint	42.39	-83.18	Retail Paints	X	13415 Lyndon St	Detroit	Michigan	48227
Sherwin-Williams Paint Store	42.41	-82.93	Retail Paints	X	17100 E Warren Ave	Detroit	Michigan	48224
Sherwin-Williams Product Finishes F	42.45	-83.18	Retail Paints	X	13101 Northend Ave	Oak Park	Michigan	48237
Smiley Paint Supply	42.33	-83.29	Retail Paints	X	25248 Ford Rd	Dearborn Heights	Michigan	48127
1-800-Cars4Cash	42.40	-83.18	Scrap	Scrap metals	13840 Fenkell Ave.	Detroit	Michigan	48227
A & Z Scrap	42.34	-83.23	Scrap	Scrap metals	19550 West Warren	Detroit	Michigan	48228
A Gold & Sons			Scrap	Scrap metals		Detroit	Michigan	48201
Abdul's Trucking	42.42	-83.10	Scrap	Scrap metals	129 Candler St	Highland Park	Michigan	48234
Cash for junk cars in Detroit mi	42.44	-83.06	Scrap	Scrap metals	19714 Ryan Rd	Detroit	Michigan	48234
Commodity Resources	42.37	-83.03	Scrap	Scrap metals	3433 E Warren Ave	Detroit	Michigan	48207
Continental Metal Co Inc	42.40	-83.07	Scrap	Scrap metals	11500 Russell St	Detroit	Michigan	48211
Detroit Iron & Metal Co	42.32	-83.13	Scrap	Scrap metals	8300 Dix Ave	Detroit	Michigan	48209
Detroit Iron & Metal Company	42.35	-83.07	Scrap	Scrap metals	Detroit		Michigan	48201
Downriver Torching Service	42.28	-83.14	Scrap	Scrap metals	750 S Deacon St	Detroit	Michigan	48217
Fast Cash for your Junk Car	42.37	-83.18	Scrap	Scrap metals	9960 Freeland	Detroit	Michigan	48227
Freedman David Inc	42.29	-83.16	Scrap	Scrap metals	893 S Dix St	Detroit	Michigan	48217
Fritz Enterprises	42.37	-83.15	Scrap	Scrap metals	7777 W Chicago	Detroit	Michigan	48204
Global Titanium Inc	42.44	-83.04	Scrap	Scrap metals	19300 Filer St	Detroit	Michigan	48234
GLR Advanced Recycling - Cars	42.40	-83.18	Scrap	Scrap metals	13840 Fenkell	Detroit	Michigan	48227
H & H Metals	42.29	-83.33	Scrap	Scrap metals	29001 Michigan Ave	Inkster	Michigan	48141
Iron Mike's Metal Prices USA LLC	42.38	-83.20	Scrap	Scrap metals	12844 Greenfield Rd	Detroit	Michigan	48227
Junk Car Top Dollar	42.33	-83.19	Scrap	Scrap metals	6031 Coleman St	Dearborn	Michigan	48126
Junk My Car Detroit	42.40	-83.18	Scrap	Scrap metals	13840 Fenkell Avenue	Detroit	Michigan	48227
Kee Container Services LLC	42.41	-83.04	Scrap	Scrap metals	13200 Mount Elliott St	Hamtramck	Michigan	48212
Kimmel Scrap Iron & Metal	42.37	-83.15	Scrap	Scrap metals	10571 Grand River	Detroit	Michigan	48204
MAG Automotive Recycling and Cas	42.43	-83.04	Scrap	Scrap metals	18489 Mt Elliot	Detroit	Michigan	48234
McNichols Scrap Iron & Metal Co			Scrap	Scrap metals		Detroit	Michigan	48201
Metal Dynamics Detroit	42.32	-83.14	Scrap	Scrap metals	3100 Lonyo St	Detroit	Michigan	48209
Metro Detroit Metals LLC	42.40	-83.07	Scrap	Scrap metals	1401 Woodland St	Detroit	Michigan	48211
Midwest Steel	42.29	-83.16	Scrap	Scrap metals	13500 Pleasant St	Detroit	Michigan	48217
Parts A Plenty Inc	42.40	-83.16	Scrap	Scrap metals	10441 Lyndon St	Detroit	Michigan	48238
Pita Metals	42.29	-83.15	Scrap	Scrap metals	411 S Fort St	Detroit	Michigan	48217
Pro Green Recycling	42.40	-83.28	Scrap	Scrap metals	15500 Telegraph Rd	Redford	Michigan	48239
PSC Metals	42.42	-83.04	Scrap	Scrap metals	6450 E McNichols Rd	Hamtramck	Michigan	48212
Rex Meadows	42.36	-83.19	Scrap	Scrap metals	9151 Hubbell St	Detroit	Michigan	48228
Rex Metal Recycling Company	42.36	-83.19	Scrap	Scrap metals	9151 Hubbell St	Detroit	Michigan	48228
RLB Car Care	42.36	-83.15	Scrap	Scrap metals	8951 Alpine St	Detroit	Michigan	48204
Scrap Metal Management	42.32	-83.13	Scrap	Scrap metals	7900 Dix St	Detroit	Michigan	48209
Scrap Metal Processing	42.32	-83.13	Scrap	Scrap metals	7900 Dix St	Detroit	Michigan	48209
Sikora Metals Inc	42.35	-83.07	Scrap	Scrap metals		Detroit	Michigan	48201
Silver's Metal Co			Scrap	Scrap metals		Detroit	Michigan	48201
Smile Recycling			Scrap	Scrap metals		Detroit	Michigan	48201
Southwest Metals Inc	42.30	-83.12	Scrap	Scrap metals	8122 W Fort St	Detroit	Michigan	48209
State Metal Co	42.30	-83.09	Scrap	Scrap metals	444 S Campbell St	Detroit	Michigan	48209
Tri Star International Trading Co	42.38	-83.20	Scrap	Scrap metals	15750 Fullerton St	Detroit	Michigan	48227
Vito's Salvage	42.42	-83.04	Scrap	Scrap metals	6440 E Davison St	Hamtramck	Michigan	48212
We Buy Junk Cars	42.37	-83.18	Scrap	Scrap metals	14301 W Chicago St	Detroit	Michigan	48228
Winston Brothers Iron & Metal Co	42.42	-83.07	Scrap	Scrap metals	17384 Conant Street	Detroit	Michigan	48212
Cardinal Recycling	42.42	-83.04	Scrap	Scrap metals	6401 E Davison St	Hamtramck	Michigan	48212
City Recycling Inc	42.35	-83.04	Scrap	Scrap metals	1943 Mack Ave	Detroit	Michigan	48207
Detroit Iron & Metal Co	42.32	-83.13	Scrap	Scrap metals	8300 Dix Ave	Detroit	Michigan	48209
Ferrous Processing & Trading Co	42.32	-83.15	Scrap	Scrap metals	9100 John Kronk St	Detroit	Michigan	48210
Ferrous Processing & Trading Co Kro	42.32	-83.15	Scrap	Scrap metals	9100 John Kronk St	Detroit	Michigan	48210
Ferrous Processing & Trading Lonyo	42.32	-83.14	Scrap	Scrap metals	3100 Lonyo St	Detroit	Michigan	48209
Fort Iron & Metal	42.29	-83.13	Scrap	Scrap metals	9607 Dearborn St	Detroit	Michigan	48209

GLE Scrap Metal - Detroit	42.27	-83.17	Scrap	Scrap metals	25435 W Outer Dr	Melvindale	Michigan	48122
GLE Scrap Metal - Warren	42.46	-83.03	Scrap	Scrap metals	22200 Sherwood Ave	Warren	Michigan	48091
GT Michigan Scrap Recycling	42.45	-83.09	Scrap	Scrap metals	1411 E 8 Mile Service Rd	Hazel Park	Michigan	48030
Kimmel Scrap Iron & Metal Co	42.37	-83.15	Scrap	Scrap metals	10571 Grand River Ave	Detroit	Michigan	48204
Lafayette Recycling	42.32	-83.13	Scrap	Scrap metals	7700 Dix St	Detroit	Michigan	48209
McNichols Scrap Iron & Metal	42.42	-83.04	Scrap	Scrap metals	6500 E McNichols Rd	Detroit	Michigan	48212
Pita Metals	42.29	-83.15	Scrap	Scrap metals	411 S Fort St	Detroit	Michigan	48217
Red Metals Recycling	42.37	-83.28	Scrap	Scrap metals	12065 Telegraph Rd	Redford Charter Twp	Michigan	48239
Schlafer Iron & Steel Inc	42.37	-83.05	Scrap	Scrap metals	1950 Medbury St	Detroit	Michigan	48211
Scrap Recyclers LLC	42.45	-83.09	Scrap	Scrap metals	1201 8 Mile Rd	Hazel Park	Michigan	48030
Sikora Metals	42.41	-83.04	Scrap	Scrap metals	12850 Mt Elliott St	Detroit	Michigan	48212
SLC Recycling Industries Inc	42.45	-83.02	Scrap	Scrap metals	8701 8 Mile Rd	Warren	Michigan	48089
Southend Metals Recycling	42.32	-83.16	Scrap	Scrap metals	3740 Wyoming Ave	Dearborn	Michigan	48120
Strong Steel Products	42.38	-83.03	Scrap	Scrap metals	6464 Strong St	Detroit	Michigan	48211
Winston Brothers Iron & Metal Comp	42.42	-83.06	Scrap	Scrap metals	17384 Conant	Detroit	Michigan	48212
A & B Steel Shearing Inc	42.44	-83.04	Steel Products and Processing	Metal fabricator	3840 Outer Dr E	Detroit	Michigan	48234
Aaple fabricating and Conveyors	42.44	-83.04	Steel Products and Processing	Metal fabricator	19354 Mt Elliott St	Detroit	Michigan	48234
Alro Steel Processing, Detroit Kurtz	42.43	-83.03	Steel Products and Processing	Metal fabricator	18881 Sherwood St	Detroit	Michigan	48234
Chappell Steel Co Inc	42.33	-83.10	Steel Products and Processing	Metal fabricator	3545 Scotten St	Detroit	Michigan	48210
Concentric Steel	42.43	-83.06	Steel Products and Processing	Metal fabricator	4150 Nevada Ave	Detroit	Michigan	48234
Detroit Steel Processing	42.38	-83.19	Steel Products and Processing	Metal fabricator	12301 Hubbell Ave	Detroit	Michigan	48227
Finkl Steel - Composite	42.32	-83.07	Steel Products and Processing	Metal fabricator	2323 W Fort St	Detroit	Michigan	48216
Gal-Cro Steel Processing	42.33	-83.13	Steel Products and Processing	Metal fabricator	3631 Parkinson St	Detroit	Michigan	48210
Hascall Steel Company	42.39	-83.04	Steel Products and Processing	Metal fabricator	6349 Strong St	Detroit	Michigan	48211
Koil Metals	42.35	-83.01	Steel Products and Processing	Metal fabricator	3400 E Lafayette St	Detroit	Michigan	48207
Liberty Steel	42.40	-82.99	Steel Products and Processing	Metal fabricator	11100- 11148 Harper Ave	Detroit	Michigan	48213
Midwest Steel Inc	42.38	-83.05	Steel Products and Processing	Metal fabricator	2525 E Grand Blvd	Detroit	Michigan	48211
Mill Steel Company - Melvindale	42.28	-83.17	Steel Products and Processing	Metal fabricator	18030 Rialto St	Melvindale	Michigan	48122
Olympic Steel	42.33	-83.12	Steel Products and Processing	Metal fabricator	3600 Military St	Detroit	Michigan	48210
Pacific Steel	42.42	-83.02	Steel Products and Processing	Metal fabricator	17157 Van Dyke Ave	Detroit	Michigan	48234
Security Steel Processing Co	42.38	-83.19	Steel Products and Processing	Metal fabricator	12211 Coyle Ave	Detroit	Michigan	48227
Service Steel	42.42	-83.03	Steel Products and Processing	Metal fabricator	13700 Sherwood St	Detroit	Michigan	48212
SMI STEEL LLC	42.42	-83.03	Steel Products and Processing	Metal fabricator	6773 E Davison St	Detroit	Michigan	48212
The Federal Group USA	42.45	-83.17	Steel Products and Processing	Metal fabricator	10711 Northend Ave	Ferndale	Michigan	48220
Tri-Star Steel Processing Center - A	42.36	-83.18	Steel Products and Processing	Metal fabricator	14334 Ellis St	Detroit	Michigan	48228
Unique Metal Products	42.46	-83.12	Steel Products and Processing	Metal fabricator	1300 Hilton Rd	Ferndale	Michigan	48220
V & S Detroit Galvanizing LLC	42.38	-83.29	Steel Products and Processing	Metal fabricator	12600 Arnold St	Redford Charter Twp	Michigan	48239
Addison Iron Fabricators	42.35	-83.02	Welding	Welding	3449 E Vernor Hwy	Detroit	Michigan	48207
Airgas Fill Plant	42.46	-83.12	Welding	Welding	1200 Farrow St	Ferndale	Michigan	48220
American Welding & Press Rpr	42.44	-83.31	Welding	Welding	26500 Eight Mile Rd	Southfield	Michigan	48033
Ayoub Custom Welding	42.34	-83.14	Welding	Welding	7525 W Warren Ave	Detroit	Michigan	48210
Carter's Welding	42.29	-83.17	Welding	Welding	14300 Mellon St	Detroit	Michigan	48217
CBP Fabrication	42.38	-83.20	Welding	Welding	12700 Mansfield St	Detroit	Michigan	48227
Classic Welding Inc.	42.45	-83.06	Welding	Welding	21500 Ryan Rd	Warren	Michigan	48091
Conner's Welding Inc	42.41	-83.02	Welding	Welding	8081 Lynch Rd	Detroit	Michigan	48234
DC Mobile Welding			Welding	Welding		Detroit	Michigan	48272
Downriver Welding	42.28	-83.17	Welding	Welding	17580 Dix Rd	Melvindale	Michigan	48122
Dunn's Welding Inc	42.46	-83.26	Welding	Welding	22930 Lahser Rd	Southfield	Michigan	48033
Electro Arc Welding	42.43	-83.05	Welding	Welding	5717 E 7 Mile Rd	Detroit	Michigan	48234
Fairlane Welding	42.35	-83.19	Welding	Welding	14711 Tireman Ave	Dearborn	Michigan	48126
Fav's Welding Services	42.28	-83.16	Welding	Welding	17126 Francis St	Melvindale	Michigan	48122
Ferrous Wolf Fabrication	42.37	-83.02	Welding	Welding	4801 Bellevue St	Detroit	Michigan	48207
Gutes Welding	42.29	-83.15	Welding	Welding	779 S Dumfries St	Detroit	Michigan	48217
Imperial Welding Inc	42.43	-83.30	Welding	Welding	25838 W 7 Mile Rd	Redford	Michigan	48240
Jams Metal Fab	42.40	-83.02	Welding	Welding	8700 Grinnell St	Detroit	Michigan	48213
Jeford Industries	42.31	-83.08	Welding	Welding	4120 W Jefferson Ave	Detroit	Michigan	48209
John E Green Co	42.41	-83.09	Welding	Welding	200 Victor St	Highland Park	Michigan	48203
Just In Time Welding LLC	42.36	-82.98	Welding	Welding	385 Parkview Dr	Detroit	Michigan	48214
King's Welding Works Inc	42.46	-83.17	Welding	Welding	10311 Capital St	Oak Park	Michigan	48237
L & B Welding	42.31	-83.15	Welding	Welding	3601 Wyoming St	Dearborn	Michigan	48120
Luckys Welding	42.29	-83.16	Welding	Welding	895 S Dix St	Detroit	Michigan	48217
Lynch Welding Company	42.41	-83.02	Welding	Welding	8081 Lynch Rd	Detroit	Michigan	48234
Marathon Metals LLC	42.36	-83.02	Welding	Welding	6440 Mack Ave	Detroit	Michigan	48207
Master Welding	42.40	-83.02	Welding	Welding	9425 Van Dyke St	Detroit	Michigan	48213
Mc Hugh Iron & Steel Corporation	42.33	-83.08	Welding	Welding	2400 20th St	Detroit	Michigan	48216
Metro Corp	42.42	-83.06	Welding	Welding	17385 Ryan Rd	Hamtramck	Michigan	48212
Michigan Welding Services	42.27	-83.30	Welding	Welding	26508 Powers Ave	Dearborn Heights	Michigan	48125
Mobile master welding	42.40	-83.02	Welding	Welding	9425 Van Dyke Ave	Detroit	Michigan	48213
Monroe Mobile Welding	42.26	-83.15	Welding	Welding	2977 S Bassett St	Detroit	Michigan	48217

National Tool & Die Welding Co	42.38	-83.35	Welding	Welding	13340 Merriman Rd	Livonia	Michigan	48150
Northern Tier Services Inc	42.41	-83.02	Welding	Welding	8081 Lynch Rd	Detroit	Michigan	48234
Plumbing & Heating Industry	42.44	-83.19	Welding	Welding	14801 W 8 Mile Rd	Detroit	Michigan	48235
Premier Rack & Fabricating	42.38	-83.28	Welding	Welding	24518 Capitol	Redford	Michigan	48239
Prima Welding & Experimental, Inc.	42.52	-82.95	Welding	Welding	31000 Fraser Dr	Fraser	Michigan	48026
Purvis and Foster, Inc.	42.40	-83.01	Welding	Welding	9640 Grinnell	Detroit	Michigan	48213
Quality Fab & Vent Inc	42.41	-83.15	Welding	Welding	8400 Puritan St	Detroit	Michigan	48238
Quality Welding & Fabrication	42.34	-83.23	Welding	Welding	6358 Grandville Ave	Detroit	Michigan	48228
R & B Welding	42.42	-83.07	Welding	Welding	3415 E McNichols Rd	Hamtramck	Michigan	48212
Sams Welding	42.34	-83.17	Welding	Welding	6903 Hartwell St	Dearborn	Michigan	48126
Sav's Welding Services, Inc	42.28	-83.14	Welding	Welding	11811 Pleasant St	Detroit	Michigan	48217
Smith	42.43	-83.04	Welding	Welding	18508 Albany St	Detroit	Michigan	48234
Starlite Welding Inc	42.37	-83.28	Welding	Welding	12091 Woodbine Ave	Redford Charter Twp	Michigan	48239
Tipaloy Inc	42.37	-83.06	Welding	Welding	1435 E Milwaukee Ave St	Detroit	Michigan	48211
Weldtech	42.32	-83.16	Welding	Welding	4401 Wyoming Ave	Dearborn	Michigan	48126
Willis fabrication and custom welding	42.38	-83.17	Welding	Welding	13200 Grand River Ave	Detroit	Michigan	48227
